# Pornography Violence Exposure Scale: a graded bifactor measure of exposure to violent pornographic content

**DOI:** 10.3389/fpsyg.2026.1885321

**Published:** 2026-10-27

**Authors:** J. Francisco Estévez-García, Vanesa Pérez-Martínez, Ariadna Cerdán-Torregrosa, Carmen Vives-Cases, Laura Otero-García, Belén Sanz-Barbero

**Affiliations:** 1 Department of Sociology II, University of Alicante, Alicante, Spain; 2 Department of Community Nursing, Preventive Medicine and Public Health and History of Science, University of Alicante, Alicante, Spain; 3 Consortium for Biomedical Research in Epidemiology & Public Health (CIBERESP), Madrid, Spain; 4 Department of Epidemiology and Statistics, National School of Public Health, Instituto de Salud Carlos III, Madrid, Spain

**Keywords:** pornography viewing, sexual violence, psychometric properties, exposure measurement, young adults, bifactor model, measurement invariance, Spain

## Abstract

**Introduction:**

Research on associations between pornography viewing and sexual violence has been limited by the absence of validated instruments that differentiate exposure along a violence continuum within pornographic practices, with existing measures focused on consumption frequency or problematic patterns rather than on the specific content consumed. The Pornography Violence Exposure Scale (PVES) was developed to assess exposure to pornographic content graded across three violence levels: non-violent material and domination-without-pain (Level 1), coercive content (Level 2), and explicit physical aggression (Level 3).

**Methods:**

A cross-sectional study with 2,181 Spanish young adults aged 18–35 years who reported viewing pornography examined factor structure, measurement invariance across sex and age, convergent, nomological, and incremental validity, and discriminative capacity for sexual violence outcomes via Receiver Operating Characteristic (ROC) analysis.

**Results:**

A bifactor model demonstrated good fit (CFI = 0.978, TLI = 0.957, RMSEA = 0.076), with a dominant general factor (ωH = 0.84, ECV = 0.81) capturing graded exposure to violent content. Strict measurement invariance was supported across sex and age groups. Convergent correlations with sexual violence outcomes (mean |*r*| = 0.24) substantially exceeded near-zero nomological associations with sexual function and partner-evaluation indicators (mean |r| = 0.03), and the general factor produced significant incremental validity for both victimization (Δ*R*^2^ = 0.088) and perpetration (Δ*R*^2^ = 0.200), with acceptable discrimination for perpetration (AUC = 0.753).

**Discussion:**

The PVES is the first psychometrically validated instrument for measuring differentiated exposure to violent pornographic content, enabling more precise investigation of content-specific associations with sexual violence outcomes.

## Introduction

Pornographic material has evolved in response to historical changes. Since the advent of the Internet, the consumption, exposure and distribution of pornography have increased, due to its easy accessibility, anonymity, and the high availability of free online content (Habidin et al., 2016). At the same time, the widespread use of smartphones has facilitated access to pornography from a very early age (OECD, 2025). This means that pornography affects people of all ages nowadays (Binnie and Reavey, 2020). In terms of prevalence, studies show higher prevalence and frequency of viewing pornography in men compared to women (Ballester-Arnal et al., 2021; Sanz-Barbero et al., 2023a; Solano et al., 2020). Viewing online pornography in young adults reaches the highest percentages: 90% for men and 60% for women between the ages of 18 and 25 (Ballester et al., 2019; Sanz-Barbero et al., 2025a; Wright et al., 2016).

Nowadays the mainstream online pornography is characterized by focusing primarily on male pleasure, where women are represented as sexual objects, depersonalized, fragmented, and positioned primarily to satisfy male desires (Bridges et al., 2010; Fritz et al., 2020), reproducing the patriarchal hierarchy. Numerous studies have analyzed the association between pornography viewing and sexual aggression (Wright et al., 2016), sexual victimization (D'Abreu and Krahé, 2016), sexist attitudes such as hostile sexism (Sanz-Barbero et al., 2024), rape myth acceptance (Leon et al., 2025), and other behaviors or attitudes related to sexual health, such as condom use (Wright et al., 2019). The findings underscore that content type—specifically the presence of violence, coercion, or aggression—constitutes a critical dimension requiring precise measurement beyond general pornography use frequency (Marshall and Miller, 2019). Longitudinal (Ybarra et al., 2011) and meta-analytic studies suggest that pornography consumption is associated with sexual aggression, among men and women, with stronger effects observed for violent than nonviolent content (Wright et al., 2016; Burnay et al., 2022). These findings indicate that the nature of the material consumed may be a key factor in understanding aggression-related outcomes. This distinction is particularly important given evidence that self-reported perceptions of problematic pornography viewing may be influenced by moral incongruence rather than actual behavioral patterns (Grubbs et al., 2019), underscoring the need for instruments that assess specific content exposure objectively. Although some studies have identified associations between pornography use and sexually aggressive attitudes or behaviors, the existence of a direct causal relationship remains contested. Several authors have argued that the available evidence is constrained by methodological weaknesses, potential selection effects, and a lack of robust longitudinal findings (Diamond, 2009; Ferguson and Hartley, 2022; Peter and Valkenburg, 2016). In this context, Fisher and Grenier (1994) questioned the extent to which exposure to violent pornography shapes men’s sexual fantasies, attitudes, and behaviors toward women. More recently, Ferguson and Hartley (2022) found little evidence linking nonviolent pornography to sexual aggression and suggested that the small associations observed for violent pornography may reflect pre-existing individual characteristics rather than causal effects. According to social learning theory (Bandura, 1977), repeated exposure to violent sexual acts present in pornography facilitates the acquisition of attitudes and behavioral scripts that support coercion and violence against women (Waterman et al., 2022). The sexual script model from Gagnon and Simon (1973) proposes that sexual behavior is acquired and enacted through a process of scripting, in which individuals learn what constitutes normal, desirable, and socially acceptable sexual conduct. Exposure to pornography (Wright et al., 2016) and specifically to violent pornography (Sanz-Barbero et al., 2025a; Schuster et al., 2025) has been associated with the acquisition of risky sexual scripts that promote both aggressive behavior and sexual victimization (D'Abreu and Krahé, 2016). However, these associations present important nuances. First, while meta-analytic evidence robustly links pornography consumption to sexual aggression perpetration (Wright et al., 2016), theoretical pathways linking violent pornography exposure to victimization risk remain less empirically established (Gámez-Guadix et al., 2022). Second, the relationship between pornography and aggression is not linear. The Confluence Model of Sexual Aggression (Malamuth and Hald, 2017) suggests that the probability of a man engaging in sexual aggression is influenced by two distinct yet interconnected clusters of personality, behavioral, and developmental factors. According to applications of this model, pornography consumption may increase the risk of perpetration of sexual violence, particularly among adult and college-aged men who exhibit high levels of hostile masculinity and an orientation toward impersonal sex (Baer et al., 2015; Vega and Malamuth, 2007). In turn, this association has been shown to have a bidirectional component. Research indicates that men with antisocial or aggressive tendencies are more prone to consume pornography, particularly violent forms (Baer et al., 2015). Although several studies have adapted the Confluence Model to female perpetrators (Bettinelli et al., 2025), these investigations have primarily focused on the model’s core constructs, such as hostile femininity and impersonal sex. Unlike the literature on men, the role of pornography consumption within the Confluence Model has received little empirical attention among women, representing an important gap in the literature.

In contrast to the behavioral outcomes produced through cognitive-behavioral pathways, exposure to violent pornography shows weak and inconsistent associations with affective-relational outcomes such as sexual satisfaction (Sanz-Barbero et al., 2025b). Recent reviews and meta-analysis reveal heterogeneous findings, with associations ranging from small negative effects to null or context-dependent positive effects, likely reflecting moderating influences of content type, consumption patterns, relationship context, and individual characteristics (Rasmussen, 2016; Kohut et al., 2020; Wright et al., 2016).

However, one of the challenges that has been found when analyzing the type of pornography and the interpretation of the results of the different studies is the use of validated scales with a clear taxonomy that classify the level of violence in the pornographic content depicted. Existing validated instruments have primarily focused on assessing problematic or compulsive patterns of pornography viewing (e.g., Grubbs et al., 2010) rather than the nature of content consumed, leaving a critical measurement gap for research examining content-specific effects. Although some authors have classified the content of pornography: sexually violent material, nonviolent but sexually degrading material, nonviolent and non-degrading material, content depicting nudity without sexual activity, and material involving minors (Gunter, 2001), or have created a system based on the level of sexual explicitness: sexually suggestive vs. sexually explicit media (Leonhardt et al., 2019), they have not been empirically validated and they have not been designed to analyze the dimension of the level of violence contained in the pornographic practices. Other recent studies have a wider classification of pornographic content (Ballester-Arnal et al., 2023), but there is no comprehensive validation analysis providing information on whether the scale accurately measures these aspects and whether it is truly well-adapted to the target population.

Given the documented associations between pornography consumption and sexual violence outcomes, and the lack of validated scales with a comprehensive classification of pornographic content, there is a critical need for measurement tools that can distinguish among content types rather than treating pornography as a homogeneous category. The aim of this study was to develop and validate the Pornography Violence Exposure Scale (PVES), a multidimensional instrument designed to measure differentiated exposure to pornographic content types.

Specifically, this research pursued five complementary objectives. First, to establish the factorial structure of the PVES through systematic exploratory and confirmatory factor analyses, examining whether theoretically distinct content types across violence intensity levels emerge as empirically separable dimensions. Second, to evaluate the internal consistency reliability of both the overall scale and its specific dimensions, ensuring adequate measurement precision for research applications. Third, to examine measurement invariance of the PVES across sex and age groups to support unbiased comparative research. Fourth, to establish construct validity through convergent validity (associations with sexual violence experiences and cyber sexual aggression), nomological validity (theoretically predicted independence from sexual function and partner and self-evaluations), and incremental validity (unique predictive variance beyond established correlates). Finally, to establish evidence-based recommendations for PVES scoring approaches and provide guidance on the appropriate use of both continuous and categorical measures for future studies examining pornography-sexual violence associations.

## Materials and methods

### Participants

Cross-sectional study of the survey “Pornography viewing, sexual health and sexual violence” conducted among 3,607 women and men aged between 18 and 35, living in Spain. To calculate the sample size, we took a frequency of pornography viewing in the last 12 months of 75% in the population aged between 18 and 35 as a reference (Sanz-Barbero et al., 2023a). For an expected OR equal to 1.5, a confidence level of 95% and a power of 80%, a minimum sample size of 3,564 questionnaires was estimated, with a relative precision of 25.4%.

The questionnaire was conducted online between July 6 and August 8, 2023. The sample design, recruitment procedures, and quality control filters have been previously described (Sanz-Barbero et al., 2025a,b). Briefly, probability-based sampling was designed considering the distribution of the Spanish population by age, sex, and region of residence, with weighting coefficients applied to meet age quotas.

This study analyzes a subsample of 2,181 individuals (*M* = 26.6 years, *SD* = 5.1; 50.2% women) who reported having watched pornography in the past 12 months.

The study was conducted according to the guidelines of the Declaration of Helsinki and approved by the Ethics Committee of the Carlos III Health Institute (Exp: CEI PI 04_2023-v3) and all participants signed an informed consent beforehand.

### Measures

#### Pornography Violence Exposure Scale (PVES)

The PVES is the focal instrument validated in the present study. The complete instrument is provided in Supplementary material, section SM1. It was developed through an iterative process involving the research team’s expertise in sexuality and pornography research, consultation with academic colleagues, and pilot testing with young adults, following procedures established for the psychometric development of sexuality-related instruments in Spanish samples (Gil-Llario et al., 2021). The instrument was developed by five researchers with expertise in gender-based violence, sexual violence, and pornography use. Following its development, it underwent a qualitative validation process involving members of the target population. Initially, a focus group of nine participants aged 21 to 34 years, all of whom had academic backgrounds in the social or health sciences, completed the questionnaire and reviewed each item. The discussion focused on participants’ understanding of the items, the suitability of the terminology used, the relevance of the proposed time frame, and the organization of items into broader thematic categories. Subsequently, fifteen individuals between 18 and 35 years of age completed the questionnaire and provided individual feedback regarding the clarity and acceptability of the items. After this validation process was completed, the research team reached consensus on the final version of the instrument. The instrument was designed to assess exposure to a graded set of pornographic content categories—from material without explicit violence to explicit physical aggression—addressing methodological limitations in existing research that treats pornography as a homogeneous category or relies on undifferentiated consumption frequency measures. Online pornography was defined as “viewing sexually explicit audiovisual material (photos, videos, short films, movies) via the Internet (computer, tablet, television, or mobile phone)”. The people surveyed answered the following question “Thinking about the last 12 months, how often do you view the following types of content when you go online to watch pornography?” The PVES conceptualizes such exposure as the availability of behavioral scripts for incorporation into viewers’ sexual schemata (Gagnon and Simon, 1973). The content categories reflect the empirically documented patterns of contemporary mainstream pornography, in which violent and non-violent material frequently co-occur within the same consumption episodes (Fritz et al., 2020; Fritz and Paul, 2017). This co-occurrence has two consequences for measurement. First, an instrument restricted to violent categories would be unable to locate a respondent’s exposure to violent material relative to the rest of what they view; the PVES therefore samples the full content range, so that non-violent categories provide the reference against which exposure to coercive and violent content is scaled. Second, the items ask how often each type of content is viewed when the respondent goes online to watch pornography, and not how often it is deliberately sought. Responses thus index the content encountered in the respondent’s habitual viewing, whether selected or incidental, which is the exposure relevant to the availability of behavioral scripts.

For the purposes of the PVES, violent content was operationalized as the explicit on-screen depiction of acts that involve at least one of three features: (a) physical aggression toward a sexual partner (e.g., hair pulling, slapping, hitting, asphyxiation); (b) the infliction of pain through implements or restraints (e.g., whipping, BDSM-related practices); or (c) sexual interactions involving force, coercion, or non-consent. Two boundaries of this definition should be made explicit. The classification is based on the act depicted and not on the consent framework portrayed around it: a scene showing pain infliction is coded as such whether or not it is framed as negotiated, because the construct of interest is the availability of the depicted act as a behavioral script, not the viewer’s judgment of its legitimacy. For the same reason, coercive depictions are counted regardless of the depicted outcome, that is, regardless of whether the person subjected to force is shown as distressed, neutral, or ultimately experiencing pleasure; the corresponding item states this explicitly (“non-consensual sex, with force, even though both end up experiencing pleasure”; SM1). Content that explicitly depicts consent negotiation is not identified by the PVES, and its potential role as a source of positive sexual scripts is a question for future instrument development rather than one the present scale can address. Accordingly, the unit of analysis is the act portrayed on screen, not the gender configuration of the depicted participants.

The PVES consists of 10 thematic blocks measuring exposure frequency to specific pornographic content categories: (1) nudity without sexual activity, (2) consensual sexual relations, (3) domination/submission without physical pain, (4) body fluid play, (5) sex with intoxicated or unconscious persons, (6) verbal threats or insults, (7) non-consensual relations with force, (8) physical aggression (hair pulling, slapping, hitting), (9) asphyxiation, and (10) BDSM with pain-inflicting implements. The blocks were derived in three steps. Categories of aggression and degradation were first drawn from content analyses of mainstream pornography (Bridges et al., 2010; Fritz et al., 2020; Fritz and Paul, 2017), which identify hair pulling, slapping, choking, verbal insults, and body fluid practices as the most frequent forms. Coercive categories (blocks 5–7) were then defined to mirror the three coercive tactics of the SAV-S (Krahé and Berger, 2013), the outcome measure used in the validation: exploitation of incapacitation, verbal pressure or threat, and physical force. Each block contains multiple sub-items differentiated by the sex composition and role of the depicted participants (e.g., man-woman, man–man, woman-woman configurations), yielding 38 sub-items total. Within each block, sub-items were summed across actor-gender configurations, so block scores capture overall exposure to the content category rather than exposure to any specific configuration of depicted participants.

For descriptive and analytical purposes, the 10 blocks were classified into three levels reflecting a graded structure of depicted violence rather than a continuous severity score. This stratification distinguishes coercive from physically violent acts, paralleling the operational distinction between psychological/coercive and physical forms applied in administrative records of intimate partner violence in Spain (Sanz-Barbero et al., 2023b), and is consistent with content-analytic taxonomies that separate degradation, coercion, and physical aggression as distinct script families (Bridges et al., 2010; Fritz and Paul, 2017).

Level 1 (blocks 1–4) covers pornographic practices without violent content as defined above. This level groups depictions of nudity, consensual sexual relations, and domination/submission practices without physical pain or coercion. While degrading scripts may be present, the depicted acts do not meet the criteria for violence in the operationalization adopted here.

Level 2 (blocks 5–7) groups coercive depictions characterized by force, coercion, or non-consent without explicit physical aggression on screen. These categories—sex with intoxicated or unconscious persons, verbal threats or insults, and non-consensual relations with force—would constitute sexual violence under most legal frameworks.

Level 3 (blocks 8–10) comprises material with explicit physical aggression or pain infliction, including physical aggression (hair pulling, slapping, hitting), asphyxiation, and BDSM with pain-inflicting implements.

Two classification decisions deserve explicit justification. First, BDSM-related practices involving implements or pain infliction (Block 10) were classified at Level 3 because the construct targets the on-screen depiction of physical pain or restraint as a script feature, independent of the negotiated consent that defines BDSM as a practiced activity. Because the items ask about content viewed when watching online pornography, responses necessarily refer to depictions as encountered by the viewer, and the item wording is behavioral (“BDSM with spanking, whips, wax, etc.”; SM1); the instrument does not ask, and cannot infer, whether a given depiction was produced within a consensual BDSM community context. Exposure to explicitly negotiated BDSM content and exposure to mainstream BDSM-like imagery therefore receive the same score, which is consistent with the construct (the depicted act) but means that the PVES should not be read as a measure of interest in or practice of BDSM. Second, body fluid play (Block 4) was classified at Level 1, that is, among the non-violent categories, because it does not meet any of the three criteria for violence defined above. Its position within Level 1 next to domination/submission (Block 3) rather than alongside nudity and consensual relations (Blocks 1–2) reflects content-analytic evidence that, in mainstream commercial pornography, it is more often embedded in degradation and objectification scripts than in mutual or affectionate ones (Bridges et al., 2010; Fritz and Paul, 2017); the items do not distinguish the affective framing of the depiction, so this placement is a statement about the typical script context of the category in mainstream content, not a classification of all such depictions as violent. Both decisions are operational choices for the construction of an exposure scale, not normative judgments about the practices themselves.

These level designations describe the type of acts depicted in pornographic content, not their legal or ethical status. The distinction between levels reflects the presence and intensity of depicted violence, not differential severity of harm.

Participants responded to each sub-item using a 5-point frequency scale, from Never (0) to Always (4), with “Prefer not to respond” coded as missing data. Because the stem is conditional on the viewing occasion, responses index the relative frequency of each content type within the respondent’s viewing, not the absolute frequency of pornography viewing.

In addition to the PVES, the following measures were administered to support construct validation through convergent, nomological, and incremental validity analyses.

#### Sexual Aggression and Victimization Scale (SAV-S)

The Sexual Aggression and Victimization Scale (SAV-S; Krahé and Berger, 2013) was used to assess sexual violence experiences. Developed as an adaptation of the behavioral approach of the Sexual Experiences Survey (Koss et al., 2007), the SAV-S employs behaviorally specific questions for both perpetration and victimization, emphasizing concrete behaviors rather than legal or interpretive labels. The scale crosses three coercive tactics (threat or use of physical force, exploitation of incapacitation, verbal pressure), four sexual behaviors (sexual touching, attempted penetration, completed penetration, other sexual acts), and three relationship contexts (current/former partner, friend/acquaintance, stranger). Cross-cultural validity has been demonstrated across multiple European samples, including Spain (Krahé et al., 2015, 2016). In the present sample, internal consistency was excellent for both victimization (*α* = 0.97, *ω* = 0.97) and perpetration (*α* = 0.99, *ω* = 0.99). For validation analyses, binary victimization and perpetration variables were derived indicating any versus no experiences in the past 12 months. The SAV-S served as convergent validity criterion in correlational analyses, as dependent variable in incremental validity testing, and as outcome variable in ROC analyses.

#### Image-based sexual abuse items

Sextortion perpetration was measured using items from the Image-Based Abuse and Victimization Scales (Gámez-Guadix et al., 2022), which assess threatening to show or publish sexual images of others without consent. The original scales have demonstrated adequate psychometric properties in Spanish youth samples. Participants reported the frequency of these behaviors in the past 12 months on a 4-point scale ranging from “Never” to “3 or more times.” In the present sample, internal consistency was good (*α* = 0.89, *ω* = 0.89; mean inter-item *r* = 0.80). These items served as convergent validity criterion, with higher pornography exposure expected to correlate positively with cyber sexual aggression (Burnay et al., 2022; Wright et al., 2016).

#### Marlowe–Crowne Social Desirability Scale (short form)

Social desirability responding was assessed using the 18-item short form of the Marlowe–Crowne Social Desirability Scale validated in Spain by Gutiérrez et al. (2016). The measure evaluates the tendency to provide socially acceptable responses. In the present sample, internal consistency was *α* = 0.64 (*ω* = 0.64), within the lower range documented in reliability generalization studies of the scale (Beretvas et al., 2002). Social desirability served as a control variable in incremental validity analyses to ensure that PVES effects were not attributable to response bias.

#### Sexual function and partner evaluation items

Constructs theoretically distinct from violent pornography exposure were assessed through five single-item measures embedded in the survey questionnaire. The analyzed items were derived from the sexual function domain of the Female Sexual Health and Dysfunctions in Primary Care instrument (items 13, 14, 15, 16, 17, and 20). This questionnaire was developed and validated in Spain by Casas Aranda et al. (2007) using a sample of women aged 18 years and older. Three items captured dimensions of sexual function—subjective arousal during sexual activity, enjoyment of sexual activity, and orgasm frequency—each rated on a 6-point response scale (1 = “Not at all” to 6 = “Very much” for arousal and enjoyment; 1 = “Never” to 6 = “Always” for orgasm frequency). Two further items captured partner evaluations: a global rating of one’s primary sexual partner and a self-rating as a sexual partner, both on a 0–10 numeric scale. These items were selected to capture experiential dimensions of personal and relational sexuality which, on theoretical grounds, depend on individual and relational factors rather than on the violent or non-violent character of consumed pornographic content (Kohut et al., 2020). They served as nomological validity criteria.

#### Demographic and control variables

Age was recorded in years and entered as a covariate in incremental validity models, with weak associations expected within the restricted 18–35 age range of the analytic sample. Sex, recorded as man or woman, was the variable on which the sampling quotas of the survey were built, and served two analytic functions: as the grouping variable for tests of measurement invariance, and as a covariate added in the third step of the incremental validity models (see Statistical Analysis).

### Statistical analysis

Analyses used complete case analysis at the level of each measure or model. For PVES validation specifically, participants were included only if they (a) reported pornography viewing in the past 12 months (*n* = 2,319) and (b) provided valid responses to all PVES sub-items, treating “prefer not to respond” as missing data rather than a separate response category. This yielded a final analytic sample of *n* = 2,181. Missing data across the total sample represented 3.83%, below established methodological thresholds at which bias from less than 5% missingness is considered trivial (Yeatts and Martin, 2015).

#### Preliminary data exploration

Prior to factor analysis, the distributional properties of the PVES were assessed to inform estimator selection. Descriptive statistics were computed for the 38 sub-items and for the 10 block scores used as observed variables in factor analysis; each block score was the sum of its constituent sub-items (3 for Blocks 1–2; 4 for Blocks 3–10). Because frequency-based behavioral measures of low-prevalence content typically depart from univariate normality (an expectation also confirmed by the present data; see Results), the robust maximum likelihood (MLR) estimator was selected for all factor analyses, providing robust inference under non-normality while preserving efficiency. Descriptive statistics and inter-block correlations are reported in Supplementary material, section SM2.

The decision to model block-level scores rather than the 38 individual sub-items reflects the conceptual structure of the PVES, in which each block represents a content category (the construct of interest), while sub-items within a block index variations in sex composition and role configuration of depicted participants (attributes of the content rather than the construct itself). This is consistent with domain-representative parceling, where aggregation across distinct facets of a common content domain is appropriate when analytical interest lies at the domain level (Hall et al., 1999; Kishton and Widaman, 1994; Little et al., 2002). Empirical support for this decision is provided in Supplementary material, section SM3.

#### Exploratory factor analysis (EFA)

Factor structure was initially examined using EFA with minimum residuals extraction on the 10 PVES block scores. Adequacy of the correlation matrix for factorization was assessed using the Kaiser–Meyer–Olkin (KMO) measure of sampling adequacy and Bartlett’s test of sphericity. Parallel analysis (100 iterations) was used to determine the number of factors to retain, and the resulting solution was rotated using geominQ (oblique) rotation to allow for factor correlations. Loadings ≥ 0.30 were considered substantively meaningful. Internal consistency reliability for the total scale and for each rotated factor was estimated using both Cronbach’s alpha and McDonald’s omega coefficients.

#### Confirmatory factor analysis and model comparison

Multiple competing models were tested via confirmatory factor analysis to determine the optimal factorial structure. Three conceptual approaches were compared: (a) a three-factor correlated model, (b) a second-order hierarchical model, and (c) a bifactor model. For models (a) and (c), baseline versions without residual correlations were estimated first and were followed by optimized versions incorporating theoretically justified residual correlations identified through modification indices (MI > 50). Models were estimated as specified in Preliminary Data Exploration. Model fit was evaluated using standard indices, with CFI and TLI ≥ 0.95 indicating excellent fit, RMSEA ≤ 0.08 acceptable fit, and SRMR ≤ 0.08 good fit (Hu and Bentler, 1999). For the bifactor models, additional indices were computed to assess the appropriateness of the bifactor structure: Explained Common Variance (ECV > 0.70), omega hierarchical (ωH > 0.70), and Percentage of Uncontaminated Correlations (PUC > 0.80). Table 1 reports fit indices for all five model specifications, and Figure 1 displays the three conceptual approaches in their optimized form.

**Table 1:** Comparative fit indices for confirmatory factor models of the PVES.

Model	*χ*^2^ scaled	df	CFI	TLI	RMSEA	SRMR	AIC	BIC
Three-factor baseline	266.8	32	0.966	0.952	0.080	0.037	85,425.8	85,556.6
Three-factor correlated	227.6	31	0.972	0.959	0.074	0.030	85,354.9	85,491.4
Second-order hierarchical	227.6	31	0.972	0.959	0.074	0.030	85,354.9	85,491.4
Bifactor original	266.1	25	0.969	0.944	0.087	0.031	85,384.5	85,555.1
Bifactor optimized	191.3	23	0.978	0.957	0.076	0.025	85,265.9	85,447.9

*χ*^2^ is the Satorra–Bentler scaled chi-square (robust under non-normality). The three-factor correlated and bifactor specifications include both baseline versions (no residual correlations) and optimized versions (with theoretically justified residual correlations identified through modification indices, MI > 50). The second-order hierarchical specification is reported in a single version, given its mathematical equivalence with the three-factor correlated optimized model when three first-order factors are specified. Specifically: Three-factor baseline = three correlated factors without residual correlations; Three-factor correlated = three correlated factors with one residual correlation (blocks 1–2); Second-order hierarchical = general factor underlying three first-order factors, with one residual correlation (blocks 1–2); Bifactor original = general factor plus three orthogonal specific factors, no residual correlations; Bifactor optimized = bifactor plus two residual correlations (blocks 1–2 and 4–5). CFI, Comparative Fit Index; TLI, Tucker–Lewis Index; RMSEA, Root Mean Square Error of Approximation; SRMR, Standardized Root Mean Square Residual; AIC, Akaike Information Criterion; BIC, Bayesian Information Criterion. Acceptable fit thresholds: CFI/TLI ≥ 0.95, RMSEA ≤ 0.08, SRMR ≤ 0.08. Lower AIC/BIC values indicate better relative fit.

**Figure 1:**
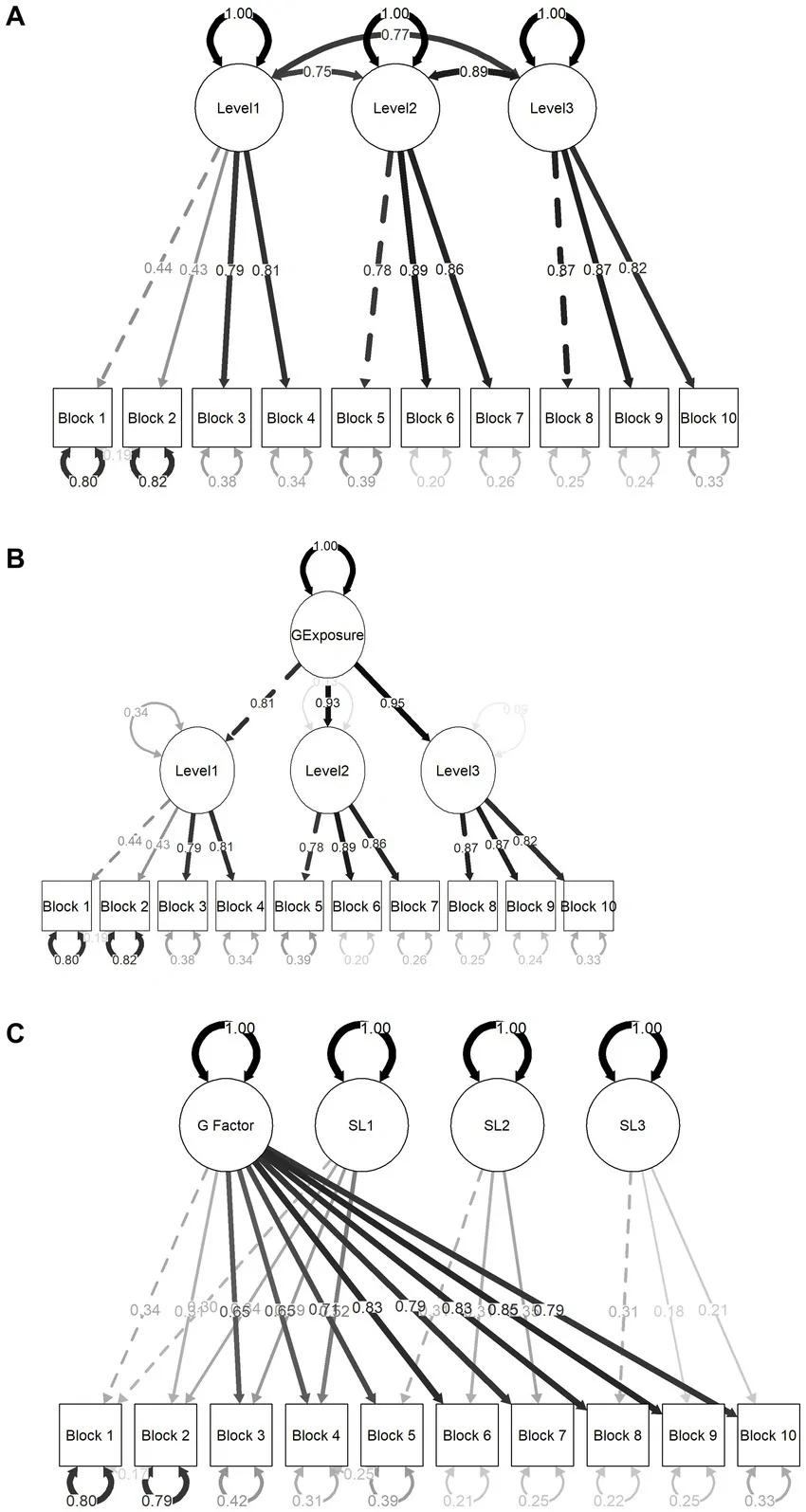
Path diagrams for the three competing models (Three Correlated Factors, Second-order, Bifactor optimized). Model **(A)**: Three correlated factors; Model **(B)**: Second-order hierarchical; Model **(C)**: Bifactor. GExposure = General Exposure; Level 1/SL1 = Level 1 (Blocks 1–4); Level 2/SL2 = Level 2 (Blocks 5–7); Level 3/SL3 = Level 3 (Blocks 8–10); G Facto*r* = General factor. Standardized loadings on paths; residual variances below variables. Dashed straight lines = weaker loadings; curved dashed lines = residual correlations. All *p <* 0.001.

To assess the robustness of the optimized bifactor structure against capitalization on chance, a 50/50 cross-validation split stratified by sex × age was conducted, with the model re-estimated independently in each half-sample under MLR. Stability across halves was evaluated through the magnitude of differences in standardized general factor loadings (|Δλ|), the consistency of bifactor-specific indices (ECV, ωH, PUC), and metric and scalar invariance tests with thresholds of ΔCFI ≥ − 0.01 and ΔRMSEA ≤ + 0.015 (Chen, 2007). Detailed results are reported in Supplementary material, section SM4.

#### Factor score extraction and scoring procedures

Following model selection, factor scores for the optimized bifactor model’s general factor were extracted using the regression method (Thurstone, 1935), implemented via *lavPredict()* in lavaan. This method produces mean-centered scores (*M* = 0) whose variance is smaller than that of the latent factor, a known property of regression-method factor scores, and is recommended for bifactor models in which the general factor captures substantial common variance (Rodríguez et al., 2016). In the present sample the general factor scores had *SD* = 0.81. Hereafter, *PVES scores* refer to these general factor scores unless otherwise specified. To inform external use of the scale when bifactor re-estimation is not feasible, Pearson correlations between the general factor scores and both (a) the simple sum across all 10 blocks and (b) within-level sums (Levels 1, 2, and 3) were computed, with bias-corrected and accelerated (BCa) bootstrap 95% confidence intervals.

### Construct validity

Construct validity was assessed through three complementary approaches following contemporary validation standards (Clark and Watson, 2019; Messick, 1995): convergent, nomological, and incremental validity.

Convergent validity examined associations between PVES scores and theoretically related constructs (sexual violence experiences and cyber sexual aggression), with positive associations expected based on meta-analytic evidence (Wright et al., 2016). Although meta-analytic support for the pornography–aggression link is robust, theoretical pathways linking violent pornography exposure to victimization remain less established (Ferguson and Hartley, 2022; Fisher and Grenier, 1994).

Nomological validity tested theoretically predicted weak or null associations with constructs outside the pornography–violence nexus (sexual function indicators, partner and self-evaluations, and age). Following Cronbach and Meehl's (1955) construct validity framework, the nomological network of a measure encompasses both relationships expected to be present and those expected to be absent or attenuated on theoretical grounds; the joint pattern of these relationships—not the magnitude of any single correlation—constitutes the validity evidence. This integrative approach is consistent with recent recommendations for comprehensive construct validation that incorporate convergent, discriminant, and incremental sources of evidence rather than relying on classical convergent–discriminant designs alone (Hunsley and Meyer, 2003).

Both convergent and nomological correlations were computed using Spearman rank coefficients with 95% confidence intervals derived from Fisher’s *z* transformation, to accommodate the non-normal distributions and ordinal measurement levels common in pornography exposure and sexual behavior data. Convergent validity was considered supported if correlations with theoretically related variables yielded meaningful positive associations whose confidence intervals excluded zero; nomological validity was considered supported if correlations with theoretically distinct constructs remained substantially lower in magnitude than convergent correlations, with many confidence intervals including zero. The ratio of mean convergent to mean nomological correlation magnitudes provided quantitative evidence for construct specificity within the nomological network framework.

Incremental validity tested whether PVES scores contributed unique predictive variance to sexual violence outcomes (victimization and perpetration as binary indicators) beyond established correlates, using a three-step hierarchical logistic regression. Model 1 included age and social desirability responding to establish a baseline. Model 2 added PVES scores to test their unique contribution. Model 3 additionally entered sex (men as the reference category) to assess whether the predictive contribution of the PVES held after controlling for participant gender. All models were fit by maximum likelihood and compared via likelihood ratio tests, with Nagelkerke *R*^2^ as the index of explained variance and Δ*R*^2^ quantifying the increment associated with each block of predictors. Statistical significance was evaluated at α = 0.05, with primary interpretation focused on effect magnitude and confidence interval precision rather than dichotomous significance testing alone.

#### ROC analysis and categorical variable development

Receiver Operating Characteristic (ROC) analysis was conducted using sexual victimization and perpetration as external binary criteria, with the bifactor general factor as the continuous predictor. Discriminative capacity was assessed via the Area Under the Curve (AUC), with 95% confidence intervals computed using DeLong’s method, and optimal cut-off points were determined through Youden’s index. Because ROC-derived cut-offs produced severely unbalanced exposure groups (see Results), a tertile-based categorization (33rd and 67th percentiles, yielding Low/Medium/High groups) was adopted, following recommendations for cases in which sensitivity–specificity-derived thresholds compromise statistical power (Schisterman et al., 2005). Discriminative validity of this categorical variable was verified via chi-square tests of independence with the sexual violence outcomes. Prevalence estimates and chi-square tests involving the categorical exposure variable were computed under the complex sampling design using survey weights, so that descriptive rates by exposure group reflected population-relevant proportions.

#### Sensitivity analyses

Two sensitivity analyses were conducted to test the robustness of the PVES general factor and to verify that its predictive contribution did not depend on the gender configuration of the depicted victim. First, the Model 2 specification described above (age, social desirability, and the focal exposure predictor) was re-estimated for both outcomes substituting the general factor with sub-scores of exposure to violence directed at female depicted victims (VAW) and at male depicted victims (VAM); each sub-score was constructed by summing sub-items within the six violent blocks (Levels 2 and 3) according to the actor configuration. Sub-scores were tested separately and jointly, with model comparisons via likelihood ratio tests. Second, the magnitude of association between each sub-score and the general factor was quantified through Pearson correlations with bias-corrected and accelerated (BCa) bootstrap 95% confidence intervals; Spearman coefficients and detailed estimates are reported in Supplementary material, section SM5.

All analyses were conducted in R (version 4.5.0; R Core Team, 2025). Key packages were lavaan (Rosseel, 2012) for confirmatory factor analysis and factor score extraction, psych for descriptive statistics and exploratory factor analysis, pROC for ROC analysis, and survey for the weighted descriptive estimates of the categorical exposure variable.

## Results

### Preliminary data exploration results

Block-score means ranged from 0.69 to 4.55 (average across blocks: *M* = 1.81), indicating heterogeneous exposure profiles. Blocks 1 and 2 (nudity without sexual activity, consensual sexual relations) showed the highest exposure rates, whereas blocks 5, 6, 7, and 9 accumulated more than 75% zero responses.

Blocks 1–4 (Level 1: nudity, consensual relations, domination without pain, body fluid play) showed relatively normal distributions (skewness < 1.6 and near-normal kurtosis), whereas blocks 5–10 (Levels 2 and 3) showed extreme positive skew (skewness > 2.4) and heavy tails (kurtosis > 7). Shapiro–Wilk tests confirmed significant departures from normality across all blocks (*p* < 0.001), as expected given the sample size, supporting the selection of MLR estimation (see Supplementary material, section SM6, for distribution plots).

### Factor structure and model selection results

#### Exploratory factor analysis

Data suitability for factor analysis was confirmed with excellent sampling adequacy (KMO = 0.920) and significant intercorrelations among variables (Bartlett’s test: *χ*^2^ = 13,231.69, *p* < 0.001). Parallel analysis supported a three-factor solution that collectively explained 62.1% of the total variance, with acceptable fit (TLI = 0.958, RMSEA = 0.075).

The three factors corresponded to the theoretical levels. Factor 1 (Level 1; 26.3% of variance) loaded on blocks 1–4: nudity without sexual activity, consensual sexual relations, domination/submission without physical pain, and body fluid play (factor loadings 0.551–0.670). Factor 2 (Level 2; 18.3%) loaded on blocks 5–7: sex with intoxicated or unconscious persons, verbal threats or insults, and non-consensual relations with force (factor loadings 0.544–0.711). Factor 3 (Level 3; 17.6%) loaded on blocks 8–10: physical aggression, asphyxiation, and BDSM with pain-inflicting implements (factor loadings 0.703–0.814).

#### Model specification and comparative evaluation

To determine the optimal factor structure of the PVES, three confirmatory factor models were specified and compared, representing different theoretical approaches to the construct (Figure 1). The first specification was a three-factor correlated model (Figure 1A), in which the 10 blocks were organized into the three theoretical levels: Level 1 (blocks 1–4), Level 2 (blocks 5–7), and Level 3 (blocks 8–10). The second was a second-order hierarchical model (Figure 1B), in which a single general factor underlies the three first-order level factors. The third was a bifactor model (Figure 1C), in which a general factor saturates all 10 blocks and three orthogonal specific factors capture variance unique to each level.

For the three-factor correlated and bifactor specifications, baseline versions without residual correlations were estimated first and then followed by optimized versions incorporating theoretically justified residual correlations identified through modification indices (MI > 50). The second-order specification was estimated in a single version, since with three first-order factors a second-order factor is just-identified and reduces to the three-factor correlated model. Table 1 reports fit indices for all five resulting model specifications.

As anticipated by the just-identification of the second-order factor with three first-order factors, the second-order and three-factor correlated models yielded identical fit indices (RMSEA = 0.074, CFI = 0.972, TLI = 0.959, SRMR = 0.030). The three-factor correlated solution further exhibited substantial inter-factor correlations (Level 2 with Level 3, *r* = 0.89; Level 1 with Level 3, *r* = 0.77; Level 1 with Level 2, *r* = 0.75), with the Level 2–Level 3 correlation reflecting severe collinearity that complicates the interpretability and stability of subsequent multivariate analyses.

#### Bifactor model selection and optimization

The bifactor specification was selected as the primary candidate because the orthogonality between general and specific factors directly addresses the inter-factor collinearity observed in the three-factor correlated solution. The bifactor original (no residual correlations) showed mixed fit (CFI = 0.969, TLI = 0.944, RMSEA = 0.087), warranting optimization.

Examination of modification indices identified two theoretically justifiable residual correlations. The first, between blocks 1 and 2 (nudity without sexual activity, consensual sexual relations; MI > 50), was the same residual correlation incorporated in the three-factor correlated optimized model and reflects the close conceptual proximity of those two non-violent categories. The second, between blocks 4 and 5 (body fluid play, sex with intoxicated or unconscious persons; MI = 71.4), was justified by their shared scripts of degradation and sexual objectification, content that the level factors do not capture in full.

Incorporation of these residual correlations yielded substantial fit improvement: CFI = 0.978, TLI = 0.957, RMSEA = 0.076, SRMR = 0.025. Bifactor-specific indices supported the appropriateness of the structure: ECV = 0.81 (the general factor accounts for 81% of common variance), ωH = 0.84 (adequate reliability of the general factor net of specific factors), and PUC = 0.73. The slightly below-threshold PUC is offset by ECV and ωH well above their thresholds, an established compensatory pattern when dimensions are conceptually interrelated (Rodríguez et al., 2016; Luo et al., 2022).

The general factor showed adequate reliability (*ω* = 0.79), whereas specific-factor reliabilities were low (Level 1: *ω* = 0.34, Level 2: *ω* = 0.19, Level 3: *ω* = 0.11), confirming that most reliable variance resides in the general factor (Table 2).

**Table 2:** Bifactor model indices and reliability estimates for the PVES.

Index/factor	Value
ECV	0.814
ω_H_	0.844
PUC	0.733
*ω* General factor	0.79
*ω* Level 1 factor	0.34
*ω* Level 2 factor	0.19
*ω* Level 3 factor	0.11

ECV, Explained Common Variance; ωH, Omega Hierarchical; PUC, Percentage of Uncontaminated Correlations. ECV indicates the proportion of common variance explained by the general factor. ωH indicates the reliability of the general factor after controlling specific factors. PUC indicates the degree to which correlations are uncontaminated by multidimensionality.

Based on this evidence, the general factor of the optimized bifactor model was adopted as the primary measure of violent pornography exposure for subsequent analyses. The decision rested on three considerations: superior model fit, full resolution of inter-factor collinearity through orthogonalization, and interpretability as a unidimensional exposure measure. Supplementary analyses confirmed that the general factor captures violent content exposure rather than overall consumption volume: it correlated markedly more strongly with violent content blocks (*r* = 0.96 for Level 3, *r* = 0.91 for Level 2) than with Level 1 baseline content (*r* = 0.42 for nudity and consensual sexual relations combined; *r* = 0.32 for consensual sexual relations alone), and individuals with high Level 1 exposure but low Level 2 and Level 3 exposure consistently fell below the mean on the general factor (Supplementary material, section SM7).

To assess the robustness of the optimized bifactor structure against capitalization on chance, a 50/50 cross-validation split stratified by sex × age was conducted under MLR estimation (see Supplementary material, section SM4). The structure replicated stably across sub-samples: all 10 general factor loadings showed |Δλ| < 0.10, and ECV (0.81), ωH (0.84), and PUC (0.73) were virtually identical between halves. Both metric and scalar invariance were supported (ΔCFI ≤ 0.003, ΔRMSEA ≤ 0.006).

### Measurement invariance

The optimized bifactor model demonstrated strict measurement invariance across sex and age groups (Table 3). All four sequential comparisons (configural → metric → scalar → strict) met Chen's (2007) thresholds: for sex, ΔCFI ranged from −0.002 to −0.009 and ΔRMSEA from −0.008 to +0.006; for age, ΔCFI ranged from −0.003 to 0.000 and ΔRMSEA from −0.007 to −0.002. In some constrained models RMSEA improved over the unconstrained baseline, indicating that the equality constraints were consistent with the underlying data structure.

**Table 3:** Measurement invariance analysis across sex and age groups.

Variable	Model	*χ* ^2^	df	CFI	TLI	RMSEA	SRMR	Δ CFI	Δ RMSEA	Invariance Support
Sex	Configural	217.89	46	0.98	0.96	0.076	0.025	—	—	—
	Metric	233.88	62	0.98	0.96	0.068	0.036	−0.003	−0.008	Yes
	Scalar	246.01	68	0.97	0.96	0.068	0.039	−0.002	0.000	Yes
	Strict	274.02	78	0.96	0.96	0.074	0.055	−0.009	0.006	Yes
Age	Configural	217.12	46	0.98	0.96	0.075	0.023	—	—	—
	Metric	244.58	62	0.98	0.97	0.068	0.032	−0.002	−0.007	Yes
	Scalar	261.08	68	0.98	0.97	0.066	0.033	0.000	−0.002	Yes
	Strict	251.11	78	0.97	0.97	0.064	0.036	−0.003	−0.002	Yes

*N*: 2,181. CFI, Comparative Fit Index; TLI, Tucker-Lewis Index; RMSEA, Root Mean Square Error of Approximation; SRMR: Standardized Root Mean Square Residual. Age groups: younger adults (18–29 years, *n*: 1,137), older adults (30–35 years, *n*: 1,044). Invariance support based on Chen (2007) criteria: ΔCFI ≥ − 0.01 and ΔRMSEA ≤ + 0.015.

### Validity assessment results

Construct validity was assessed through three complementary approaches: convergent, nomological, and incremental validity (Table 4).

**Table 4:** Correlations between PVES general factor and validation criteria.

Criteria	Variable	*r*	95% CI	*p*	Type
Convergent validity	VS partner	0.23	[0.19, 0.27]	< 0.001	Convergent
	VS friend	0.25	[0.21, 0.29]	< 0.001	Convergent
	VS stranger	0.22	[0.18, 0.26]	< 0.001	Convergent
	SA partner	0.26	[0.22, 0.30]	< 0.001	Convergent
	SA friend	0.26	[0.22, 0.30]	< 0.001	Convergent
	SA stranger	0.24	[0.20, 0.28]	< 0.001	Convergent
	Sextortion threats (show)	0.21	[0.17, 0.25]	< 0.001	Convergent
	Sextortion threats (publish)	0.22	[0.18, 0.26]	< 0.001	Convergent
Nomological validity	Age	−0.06	[−0.10, −0.01]	0.009	Nomological
	Sexual arousal	−0.01	[−0.06, 0.04]	0.609	Nomological
	Sexual enjoyment	0.01	[−0.04, 0.05]	0.832	Nomological
	Orgasm frequency	−0.01	[−0.06, 0.04]	0.608	Nomological
	Partner evaluation	−0.07	[−0.11, −0.02]	0.003	Nomological
	Self-evaluation	0.02	[−0.02, 0.07]	0.350	Nomological
Response bias	Social desirability	−0.16	[−0.20, −0.11]	< 0.001	Response Bias

Spearman correlations with 95% confidence intervals. *n* = 2,181 for all analyses except sextortion variables (*n* = 2,168–2,169) and sexual function, partner-evaluation, and self-evaluation variables (*n* = 1,880–2,037). Sample sizes for sexual function, partner-evaluation, and self-evaluation variables reflect conditional survey branching: sexual arousal, enjoyment, and orgasm frequency items (*n* = 1,880) were administered only to participants reporting current sexual activity; partner evaluation and self-evaluation items (*n* = 2,037) were administered only to participants who reported lifetime sexual practices with another person. Convergent validity: mean |r| = 0.24; nomological validity: mean |r| = 0.03; ratio = 8.2:1.

Convergent validity was strongly supported across theoretically related constructs. Sexual violence experiences correlated robustly with PVES scores: victimization correlations ranged from *r* = 0.22 to *r* = 0.25 across relationship contexts (partner, friend, stranger), and sexual perpetration correlations were comparable (*r* = 0.24 to *r* = 0.26), with the strongest associations observed for partner and friend contexts. Cyber sexual perpetration behaviors (sextortion threats) yielded correlations of *r* = 0.21 to *r* = 0.22. All convergent correlations were statistically significant (95% CIs excluded zero), with mean |*r*| = 0.24.

Theoretically distinct constructs within the nomological network showed weak or null associations with PVES scores, as predicted. Sexual function indicators demonstrated near-zero correlations (arousal: *r* = −0.01; enjoyment: *r* = 0.01; orgasm frequency: *r* = −0.01; all *p* > 0.05). Partner and self-evaluations also showed minimal associations (*r* = −0.07 and *r* = 0.02, respectively); only the partner-evaluation correlation reached statistical significance, with a magnitude that is substantively negligible. Age correlated weakly and negatively (*r* = −0.06, *p* = 0.009), consistent with slight cohort differences but substantively negligible. Across nomological indicators, mean |*r*| = 0.03, with only 33% reaching statistical significance. The ratio of mean convergent to mean nomological correlations (8.2:1) provides strong evidence for construct specificity within the nomological network framework.

As expected, social desirability responding showed a moderate negative correlation with PVES scores (*r* = −0.16, *p* < 0.001), consistent with response bias patterns in which socially sensitive content elicits underreporting; the magnitude is well within acceptable ranges and does not threaten construct validity.

Incremental validity was assessed through hierarchical logistic regression testing whether PVES scores contribute unique variance to sexual violence outcomes (victimization and perpetration) beyond age and social desirability (Table 5). Model 1 included age and social desirability; Model 2 added PVES scores; Model 3 additionally entered sex (men as the reference category) (*n* = 2,181). PVES scores produced significant increments in explained variance for both outcomes: for victimization, Nagelkerke *R^2^* increased from 0.014 to 0.102 (Δ*R*^2^ = 0.088, *χ*^2^ = 132.31, *p* < 0.001); for perpetration, Nagelkerke *R^2^* increased from 0.004 to 0.205 (Δ*R*^2^ = 0.200, *χ*^2^ = 202.99, *p* < 0.001). Effect sizes were substantial in both cases: victimization OR = 2.00, 95% CI [1.76, 2.28], *p* < 0.001; perpetration OR = 2.68, 95% CI [2.32, 3.13], *p* < 0.001.

**Table 5:** Incremental validity: hierarchical logistic regression models predicting sexual violence outcomes.

Outcome	Model	Nagelkerke *R^2^*	ΔR^2^	*χ* ^2^	df	*p*
Sexual victimization	Model 1	0.014	—	—	—	—
Sexual victimization	Model 2	0.102	0.088	132.31	1	< 0.001
Sexual victimization	Model 3	0.130	0.028	43.30	1	< 0.001
Sexual perpetration	Model 1	0.004	—	—	—	—
Sexual perpetration	Model 2	0.205	0.200	202.99	1	< 0.001
Sexual perpetration	Model 3	0.206	0.001	0.90	1	0.344

*N* = 2,181. Model 1: age + social desirability. Model 2: + PVES general factor. Model 3: + sex (men as reference category). Δ*χ*^2^ statistics test each added block of predictors against the previous model. Model 2 coefficients—Sexual victimization: age (OR = 0.96, *p* = 0.002), social desirability (OR = 0.97, *p* = 0.044), PVES (OR = 2.00, *p* < 0.001); sexual perpetration: age (OR = 0.98, *p* = 0.424), social desirability (OR = 0.99, *p* = 0.628), PVES (OR = 2.68, p < 0.001). Model 3 coefficients—Sexual victimization: age (OR = 0.96, *p* = 0.010), social desirability (OR = 0.98, *p* = 0.200), PVES (OR = 2.17, *p* < 0.001), sex—women vs men (OR = 2.08, *p* < 0.001); sexual perpetration: age (OR = 0.98, *p* = 0.388), social desirability (OR = 0.98, *p* = 0.563), PVES (OR = 2.65, *p* < 0.001), sex—women vs men (OR = 0.84, *p* = 0.347).

Adding sex in Model 3 produced contrasting patterns across outcomes. For victimization, sex acted as a suppressor: the PVES odds ratio increased from 2.00 to 2.17, 95% CI [1.90, 2.50], once sex was partialed out (relative change = +8.6%; Δ*R*^2^ = 0.028, *χ*^2^ = 43.30, *p* < 0.001). Sex was independently associated with victimization [women relative to men: OR = 2.08, 95% CI (1.67, 2.58), *p* < 0.001], with women reporting more than twice the odds of victimization despite lower violent pornography exposure. For perpetration, the PVES odds ratio remained virtually unchanged [Model 3 OR = 2.65, 95% CI (2.29, 3.09); relative change = −1.2%], and sex did not contribute independent variance [women vs. men: OR = 0.84, 95% CI (0.58, 1.20), *p* = 0.347]. The PVES retains its incremental predictive validity over and above age, social desirability, and sex.

Together, the substantial gap between convergent (mean |*r*| = 0.24) and nomological correlations (mean |*r*| = 0.03), combined with robust incremental validity, supports the construct validity of the PVES as a measure of violent pornography exposure.

### ROC analysis and scale scoring

ROC analysis revealed moderate to acceptable discriminative capacity of the general factor for predicting sexual violence outcomes (Table 6; Figure 2). These analyses and the tertile-based categorization below were computed on the 2,163 participants with valid responses on both outcomes (18 participants chose not to answer one of the sexual violence items).

**Table 6:** ROC analysis and categorical variable performance for violent pornography exposure.

ROC Performance	Victimization	Perpetration
N positive cases	513	195
AUC (95% CI)	0.660 (0.633–0.688)	0.753 (0.715–0.792)
Optimal threshold	−0.133	−0.115
Sensitivity	0.558	0.713
Specificity	0.696	0.688
PPV	0.363	0.185
NPV	0.835	0.960

*N* = 2,163. AUC, Area Under the Curve with 95% confidence intervals; PPV, positive predictive value; NPV, negative predictive value.

**Figure 2:**
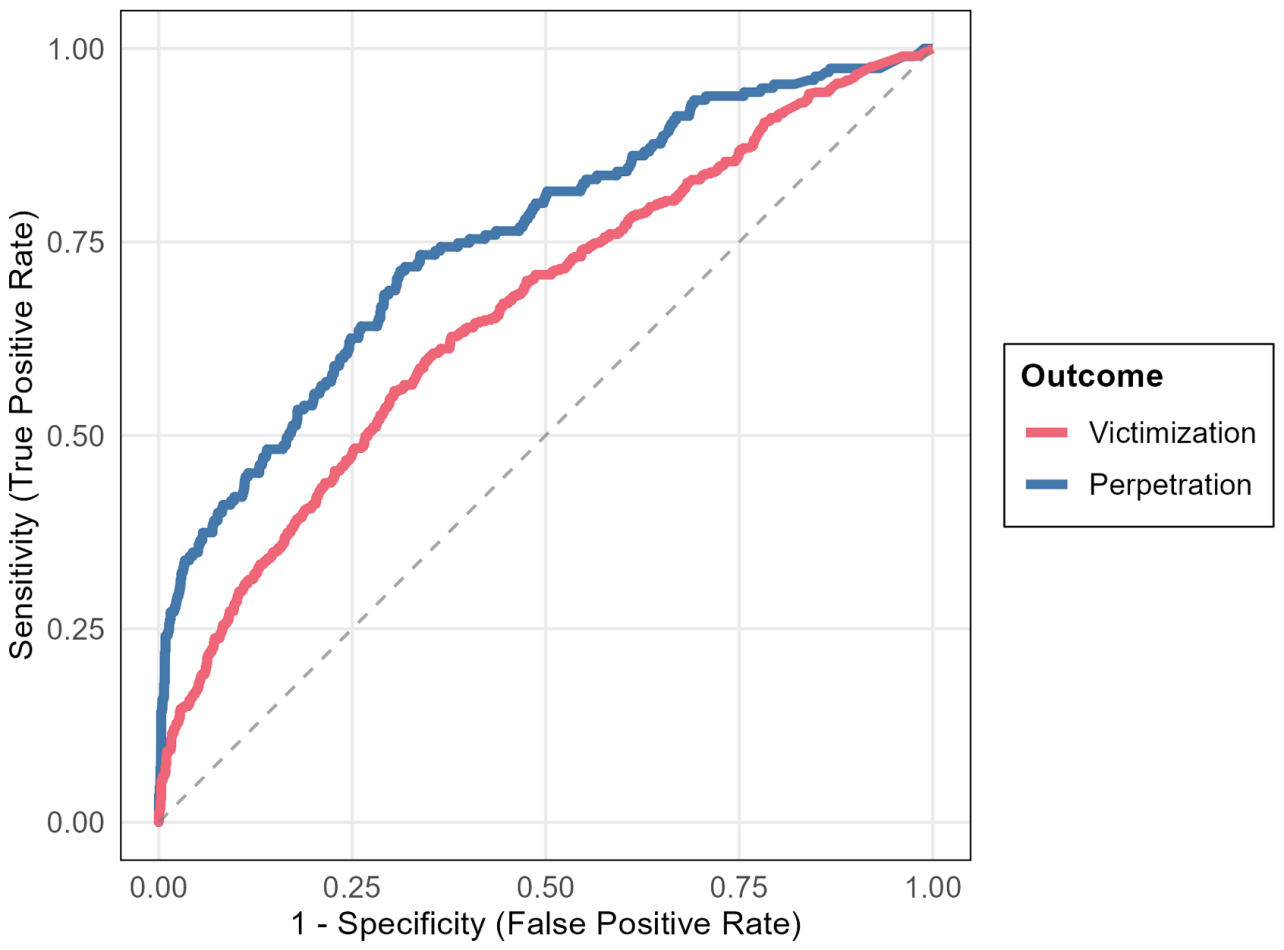
ROC curves for PVES discriminative capacity predicting sexual violence victimization and perpetration. The diagonal dashed line represents random classification (AUC = 0.50). Victimization: AUC = 0.660, 95% CI [0.633, 0.688]; Perpetration: AUC = 0.753, 95% CI [0.715, 0.792].

As expected, the general factor showed superior performance for perpetration [AUC = 0.753, 95% CI (0.715, 0.792)] compared to victimization [AUC = 0.660, 95% CI (0.633, 0.688)]. Both AUC values exceeded the threshold typically considered indicative of meaningful discriminative capacity (AUC > 0.60), with perpetration reaching the threshold for acceptable discrimination (AUC > 0.70).

Optimal cut-off points using Youden’s index were −0.133 for victimization (sensitivity = 0.558, specificity = 0.696) and −0.115 for perpetration (sensitivity = 0.713, specificity = 0.688).

#### Categorical variable development

Initial ROC-derived thresholds yielded highly unbalanced groups (1.6% in middle category), warranting an alternative categorization. Tertile-based division created three balanced exposure groups: Low (*n* = 714, 33%), Medium (*n* = 735, 34%), and High (*n* = 714, 33%). The categorization showed significant associations with both outcomes: victimization, *χ*^2^ = 99.7, *p* < 0.001; perpetration, *χ*^2^ = 121.9, *p* < 0.001.

The tertile-based classification showed clear dose–response patterns for both outcomes: victimization rates increased systematically across exposure levels (15.3%, 20.1%, and 36.0% for Low, Medium, and High, respectively), and perpetration rates followed the same pattern (3.1%, 5.4%, and 18.3%).

#### Scoring recommendations

Three scoring approaches are recommended for external use of the PVES. For psychometric research, the recommended approach is to reestimate the optimized bifactor model in a new sample and to extract general factor scores via the regression method (Thurstone, 1935); the lavaan model syntax is provided in Supplementary material, section SM8. For applied risk stratification, tertile-based classification of these factor scores follows the procedure validated in the present study. When reestimation is not feasible, sum-based scoring across the 10 blocks provides a defensible approximation: in this sample (*n* = 2,181), the simple sum correlated *r* = 0.96, BCa 95% CI [0.95, 0.97], with the general factor scores. Block-level sums correlated differently with the general factor (Level 1: *r* = 0.71; Level 2: *r* = 0.91; Level 3: *r* = 0.96), indicating that Level 3 sums closely approximate the construct, whereas Level 1 sums—which capture exposure to non-violent content—should be interpreted as a separate exposure indicator rather than as a proxy for the violence construct.

#### Sensitivity analyses: female-victim and male-victim sub-scores

To evaluate whether the general factor differently captures exposure to violence directed at female versus male depicted victims, two sub-scores were constructed by summing sub-items within the six violent blocks (Levels 2 and 3) according to the actor configuration: a female-victim sub-score (VAW; sub-items with female targets) and a male-victim sub-score (VAM; sub-items with male targets). Both sub-scores correlated very highly with the general factor scores: VAW with G, *r* = 0.96, BCa 95% CI [0.95, 0.96]; VAM with G, *r* = 0.94, BCa 95% CI [0.93, 0.95]. The two sub-scores were themselves substantially correlated, *r* = 0.84, BCa 95% CI [0.81, 0.87], reflecting a shared tendency to consume violent content irrespective of victim gender.

Hierarchical logistic regression models replicated the principal pattern of incremental validity across both outcomes. For sexual victimization, VAW alone (Nagelkerke *R^2^* = 0.100) and VAM alone (Nagelkerke *R^2^* = 0.094) yielded predictive performance comparable to that of the general factor (Nagelkerke *R^2^* = 0.102); when entered jointly, both sub-scores remained significant predictors [VAW: OR = 1.06, 95% CI (1.03, 1.09), *p* < 0.001; VAM: OR = 1.05, 95% CI (1.01, 1.08), *p* = 0.009], with a marginal improvement over the general factor model (Δχ^2^ = 4.11, *df* = 1, *p* = 0.043). For sexual perpetration, the same pattern held with greater magnitudes (VAW alone: *R^2^* = 0.202; VAM alone: *R^2^* = 0.199; combined: *R^2^* = 0.214; Δχ^2^ compared with the general factor model = 10.21, *df* = 1, *p* = 0.001). Detailed tables are provided in Supplementary material, section SM5. These results indicate that interpretations linking PVES scores to exposure to violence against women are empirically grounded, while acknowledging that the scale also captures exposure to violence against men, which itself contributes uniquely to predicting both outcomes.

## Discussion

The systematic validation of the PVES addresses a critical methodological gap in pornography research by providing the first instrument with comprehensive psychometric validation for measuring differential exposure across the full continuum from Level 1 to Level 3 pornographic content. Our findings demonstrate that PVES achieves all validation objectives, establishing a reliable and valid measure that distinguishes among theoretically distinct content types while resolving persistent measurement challenges in this research domain. Because the scale differentiates content rather than assuming a uniform effect of viewing, it also makes it possible to examine whether exposure to specific content types is associated with neutral or positive relational and educational outcomes, and whether such associations differ according to viewers’ critical engagement with the material.

The factorial structure analysis revealed that violent pornography exposure is optimally conceptualized through a bifactor model, with a dominant general factor capturing common variance across all content types and orthogonal specific factors representing unique content-type variance. This structure effectively addressed the severe multicollinearity observed in the three-factor correlated model (with the highest inter-factor correlation reaching *r = 0.89*), where traditional approaches would have compromised analytical precision. While the bifactor solution provides both a psychometrically sound general measure and theoretically interpretable specific dimensions, the limited reliability of specific factors suggests that content-type differentiation, though statistically supported, may restrict practical utility for most research applications.

The reliability evidence supports robust measurement quality, with the general factor demonstrating adequate reliability for research applications. The dominance of general factor variance, combined with limited specific factor reliabilities, confirms that the primary interpretable variance resides in the general dimension, supporting its use as the principal measure in most research contexts. Conceptually, the general factor can be interpreted as capturing graded exposure to non-consensual, coercive, and physically violent content, with Level 1 content (blocks 1–4) functioning as a reference point against which exposure to increasingly violent material is measured—a structure consistent with sexual script theory, in which exposure patterns can escalate from mainstream explicit content toward Level 2 and Level 3 material within a unified latent dimension (Gagnon and Simon, 1973). Consistent with this reading, the general factor correlated 0.91 with Level 2 sums and 0.96 with Level 3 sums but only 0.32 with consensual sexual relations (SM7), so that elevated scores reflect the coercive and violent portion of what is viewed rather than viewing as such. This hierarchical organization validates theoretical frameworks emphasizing common psychological mechanisms underlying violent pornography exposure while maintaining empirical distinctions among content types (Chen et al., 2012).

Our measurement invariance analysis achieved strict invariance across both sex and age groups, providing strong support for comparative analysis across demographic subgroups—a standard increasingly adopted in the cross-cultural validation of sexuality-related instruments (e.g., Ballester-Arnal et al., 2025). This finding indicates that the PVES measures the same construct equivalently in males and females and across age groups. Consequently, any observed group differences in PVES scores reflect genuine differences in violent pornography exposure rather than measurement bias or differential item functioning. While strict invariance supports the scale’s broad applicability, it does not imply absence of group differences; rather, it ensures that observed differences, if present, represent true construct-level variation rather than measurement artifacts.

The construct validity evidence demonstrates theoretically consistent patterns across three complementary sources: convergent validity, nomological validity, and incremental validity. Convergent correlations with sexual violence measures aligned closely with meta-analytic effect sizes linking pornography exposure to sexual aggression (Wright et al., 2016), confirming that PVES captures exposure meaningfully related to violence outcomes, consistent with script acquisition and social learning mechanisms proposed in the literature (Bandura, 1977; Gagnon and Simon, 1973). Associations spanned sexual victimization, sexual perpetration, and cyber sexual aggression, providing consistent evidence across multiple operationalizations of violence-related constructs (Wright et al., 2016).

In contrast, nomological validity testing revealed weak or null associations with theoretically distinct constructs within the broader sexual behavior domain, yielding a convergent-to-nomological ratio of 8.2:1 that provides strong quantitative evidence for construct specificity. Sexual function indicators demonstrated near-zero correlations, effectively ruling out the possibility that PVES captures positive or negative sexual experience rather than violent content exposure specifically. This pattern aligns with theoretical distinctions between cognitive-behavioral pathways (linking violent content to aggression through script learning) and affective-relational outcomes (such as sexual satisfaction), which show weak and inconsistent associations with pornography exposure across the literature (Rasmussen, 2016).

Similarly, minimal associations with partner evaluation and self-evaluation support the construct specificity of the PVES. Mainstream pornography emphasizes idealized physical attractiveness and body standards, which theoretical frameworks link to altered perceptions of partner desirability and self-evaluation as sexual partners (Paslakis et al., 2020; Tylka and Kroon Van Diest, 2015). The observed near-zero correlations suggest that PVES captures exposure to violent content characterized by aggressive acts and power dynamics rather than content primarily focused on physical appearance standards.

This pattern confirms that the scale measures progression toward violent material specifically, not mainstream pornography consumption that might influence body-image-related evaluations (Tylka and Kroon Van Diest, 2015). This pattern of selective associations supports the theoretical distinction that PVES measures type of content consumed rather than consumption frequency or volume. If the scale primarily captured general pornography viewing frequency, it would be expected to correlate broadly with various sexual outcomes regardless of content type, including positive sexual experiences and body-image evaluations (Kohut et al., 2020). As documented in the factor specificity analysis, individuals with high Level 1 but low violent consumption consistently scored below the mean on the general factor, confirming that elevated scores reflect violent content exposure rather than general consumption volume or content variety.

Incremental validity testing provided compelling evidence that PVES contributes unique predictive variance beyond established correlates. Hierarchical regression models demonstrated substantial increments in explained variance for both sexual victimization and sexual perpetration outcomes. These effect sizes confirm that violent pornography exposure, as captured by the PVES, provides predictive information beyond demographic characteristics and response bias. Sensitivity analysis additionally controlling for participant sex confirmed this robustness: the predictive contribution of the PVES to perpetration was essentially unchanged, while its contribution to victimization was preserved—and even strengthened—once sex was partialed out, in a pattern consistent with sex acting as a suppressor variable in the victimization model. This finding indicates that the predictive validity of the PVES does not rest on confounding with gender. Sub-score sensitivity analysis by victim gender further supported the interpretive grounding of the scale, with the female-victim sub-score replicating the predictive pattern of the general factor.

The stronger predictive relationship with perpetration compared to victimization, evident in the magnitude of the variance increments, aligns with the more established theoretical and empirical literature linking pornography exposure to perpetration through mechanisms such as script learning and desensitization (Wright et al., 2016) although there are also recent studies that question this association (Ferguson and Hartley, 2022). While theoretical mechanisms linking violent pornography exposure to victimization risk—such as adoption of high-risk behavioral scripts and normalization of coercive scenarios—have been proposed (Simons et al., 2012), this association remains less empirically established and represents an important finding requiring further theoretical development regarding potential pathways such as risk perception reduction. Longitudinal research is warranted to clarify these mechanisms.

The PVES demonstrated meaningful predictive utility through ROC analysis, achieving acceptable discriminative capacity for sexual violence perpetration and modest performance for victimization. The superior discriminative capacity for perpetration compared to victimization aligns with theoretical expectations and the differential evidence base (Wright et al., 2016). Active engagement with violent pornographic content operates through well-established social learning mechanisms (script acquisition, desensitization) that predict perpetration behaviors (Wright et al., 2016). In contrast, pathways linking violent content exposure to victimization risk remain theoretically underdeveloped (Rodenhizer and Edwards, 2019) and empirically less established (Simons et al., 2012), potentially explaining the moderate discriminative capacity observed. This pattern suggests that the PVES captures perpetration risk more robustly than victimization vulnerability, consistent with the stronger meta-analytic evidence for pornography-aggression associations (Burnay et al., 2022).

The empirically-derived tertile categorization provides meaningful risk stratification, with clear gradient patterns—victimization and perpetration rates increasing systematically across exposure levels. These categories, based on the validated continuous general factor, offer practical utility for group-based analysis and applied research, while retaining the discriminative capacity of the underlying continuous measure. Although the bifactor structure provides orthogonal specific factors representing content-type variance (Level 1, Level 2, Level 3), the limited reliability of these specific dimensions (*ω* = 0.11–0.34) restricts their utility for most research applications; researchers investigating differential content effects should interpret specific factor results with caution. The validation provides evidence-based scoring recommendations optimized for different research applications. The convergent-nomological-incremental validation strategy employed here provides more comprehensive construct validity evidence than classical convergent-discriminant approaches, particularly for measures of specific behavioral exposures that must demonstrate both theoretical relationships with violence outcomes and independence from related but distinct constructs within shared theoretical domains. The general factor serves as the primary measure for most analyses, particularly studies examining pornography-sexual violence associations, maximizing statistical power while avoiding multicollinearity complications. The tertile-based categorization offers specialized utility for studies requiring group-based analysis or clinical interpretation, with balanced sample sizes supporting robust between-group comparisons.

## Limitations

Several limitations warrant consideration. First, the cross-sectional design precludes causal inferences about directionality between violent pornography exposure and sexual violence outcomes; experimental or longitudinal designs are needed to establish temporal precedence.

Second, the theoretical mechanisms linking violent pornography exposure to victimization risk remain less empirically established than those for perpetration, and longitudinal designs employing mediation and path analysis are warranted to clarify these differential pathways.

Third, the sample restriction to young adults (18–35 years) who reported pornography viewing limits generalizability to broader populations and older cohorts.

Fourth, the use of a volunteer panel, despite probability-based sampling procedures, may limit generalizability due to potential self-selection bias inherent in online survey research.

Fifth, reliance on retrospective self-report may introduce recall bias.

Sixth, the scale aggregates across actor-gender configurations within each content block. While this aggregation means the PVES does not exclusively isolate violence against women, sensitivity analyses showed that a female-victim sub-score correlated *r* = 0.96 with the general factor, supporting that interpretations linking PVES scores to violence against women are empirically grounded in this sample.

A related concern is the within-block summation of sub-items across actor-gender configurations: viewers whose consumption spans multiple configurations (e.g., bisexual viewers) may obtain higher scores than viewers with equivalent intensity of violent-content exposure but a narrower range of configurations. Two features of the data limit this concern. The item stem is conditional on the viewing occasion, so scores reflect the relative frequency of each content type within a respondent’s viewing rather than the amount of pornography viewed, and the general factor correlated only moderately with self-reported frequency of online pornography viewing in the past 12 months [Spearman *ρ* = 0.34, BCa 95% CI (0.30, 0.38); Supplementary material, section SM7]. Nevertheless, future scale revisions should consider scoring procedures that control for the range of actor configurations viewed.

Seventh, a small number of respondents (*n* = 15) met at least one of three *a priori* response-quality criteria on the sexual violence items (reporting no lifetime sexual contact while endorsing perpetration, extreme straight-lining, or very short completion time; Supplementary material, section SM9). Re-estimating the convergent and incremental validity analyses without these cases left all estimates essentially unchanged.

Finally, the PVES is designed for research on population-level associations between content exposure and sexual violence outcomes. Its scores and exposure categories describe patterns of viewing, not individuals, and should not be used to characterize, screen, or label respondents.

## Conclusion

The PVES provides, to our knowledge, the first psychometrically validated instrument designed to capture differences in exposure to violent pornographic content. By moving beyond simple frequency indicators and distinguishing exposure across three graded levels of content intensity, it offers a more nuanced way to examine links between pornography exposure patterns and sexual violence experiences.

At the same time, the present findings should be interpreted within their methodological boundaries. The cross-sectional design and the focus on a Spanish young-adult sample limit the scope of the conclusions that can be drawn at this stage. Further studies in different populations and cultural contexts will be necessary to assess how well the scale generalizes, and longitudinal research, in particular, would help clarify the direction and temporal dynamics of the associations observed here.

Beyond its research value, the PVES may also have practical implications. A more precise assessment of exposure could be useful for developing educational and prevention initiatives that address the potential role of violent pornographic content in shaping sexual attitudes and behaviors.

## Data Availability

The datasets generated and analyzed during the current study are not publicly available due to ethical and privacy considerations relating to the sensitive nature of the data, but are available from the corresponding author on reasonable request.
