# Supplementary material for: Pornography Violence Exposure Scale: a graded bifactor measure of exposure to violent pornographic content
Source: Front Psychol. 2026 Oct 27;17:1885321. doi: 10.3389/fpsyg.2026.1885321 (PMC13747955; doi:10.3389/fpsyg.2026.1885321)
Supplement: Supplementary file 1 [file Supplementary_file_1.pdf]

SUPPLEMENTARY MATERIAL

Pornography Violence Exposure Scale (PVES): A Multidimensional Measure

Contents

SM1. PVES Questionnaire
SM2. Descriptive Statistics and Correlations for PVES Items and Blocks
SM3. Empirical Justification for Item Parceling
SM4. Cross-Validation of the Optimized Bifactor Model
SM5. Female-Victim and Male-Victim Sub-Score Analyses
SM6. Distribution Analysis and Normality Assessment for PVES Block Scores
SM7. Factor G Specificity Analysis: Detailed Validation Evidence
SM8. R lavaan Syntax for the Optimized Bifactor Model
SM9. Sensitivity of Criterion Validity to Response Quality

SM1. PVES Questionnaire

Thinking about the last 12 months, how often do you view the following types of content when you go online to watch pornography? *("Sexual relations" refers to activities involving vaginal or anal penetration with a penis or objects, or oral sex.)*

	Naked people without having sexual intercourse	Never	Almost never	Often	Almost always	Always	Prefer not to respond
B6_1A	Nude men and women						
B6_1B	Only men.						
B6_1C	Only women.						
	Consensual sexual relations WITHOUT THE USE OF FORCE	Never	Almost never	Often	Almost always	Always	Prefer not to respond
B6_2A	Between men and women						
B6_2B	Between men.						
B6_2C	Between women.						
	Domination and submission, WITHOUT physical pain	Never	Almost never	Often	Almost always	Always	Prefer not to respond
B6_3A	Dominant man/men – submissive woman/women						
B6_3B	Dominant man/men – submissive man/men						
B6_3C	Dominant woman/women – submissive man/men						
B6_3D	Dominant woman/women – submissive woman/women						
	PLAY involving semen and/or saliva and/or other bodily fluids on the body, face, or mouth	Never	Almost never	Often	Almost always	Always	Prefer not to respond
B6_4A	One or more men on a woman/women						
B6_4B	One or more men on a man/men						
B6_4C	One or more women on a man/men						
B6_4D	One or more women on a woman/women						
	Sexual relations with an INTOXICATED, UNCONSCIOUS, OR SLEEPING person	Never	Almost never	Often	Almost always	Always	Prefer not to respond
B6_5A	Conscious man/men – intoxicated/unconscious/sleeping woman/women						
B6_5B	Conscious man/men – intoxicated/unconscious/sleeping man/men						

B6_5C	Conscious woman/women – intoxicated/unconscious/sleeping man/men						
B6_5D	Conscious woman/women – intoxicated/unconscious/sleeping woman/women						
	Sexual relations involving verbal insults or threats	Never	Almost never	Often	Almost always	Always	Prefer not to respond
B6_6A	Man/men insult or threaten woman/women						
B6_6B	Man/men insult or threaten man/men						
B6_6C	Woman/women insult or threaten man/men						
B6_6D	Woman/women insult or threaten woman/women						
	NON-CONSENSUAL sex, WITH FORCE, even though both end up experiencing pleasure	Never	Almost never	Often	Almost always	Always	Prefer not to respond
B6_7A	Man/men force woman/women						
B6_7B	Man/men force man/men						
B6_7C	Woman/women force man/men						
B6_7D	Woman/women force woman/women						
	Sexual relations where a person PULLS HAIR, SLAPS, OR HITS another	Never	Almost never	Often	Almost always	Always	Prefer not to respond
B6_8A	Man/men toward woman/women						
B6_8B	Man/men toward man/men						
B6_8C	Woman/women toward man/men						
B6_8D	Woman/women toward woman/women						
	Sexual relations where a person CAUSES ASPHYXIATION to another	Never	Almost never	Often	Almost always	Always	Prefer not to respond
B6_9A	Man/men toward woman/women						
B6_9B	Man/men toward man/men						
B6_9C	Woman/women toward man/men						
B6_9D	Woman/women toward woman/women						
	BDSM (Bondage/Domination/Submission/Sadomasochism) with spanking, whips, wax, etc.	Never	Almost never	Often	Almost always	Always	Prefer not to respond
B6_10A	Dominant man/men – submissive woman/women						
B6_10B	Dominant man/men – submissive man/men						
B6_10C	Dominant woman/women – submissive man/men						
B6_10D	Dominant woman/women – submissive woman/women						

SM2. Descriptive Statistics and Correlations for PVES Items and Blocks

Table SM2a. *Descriptive Statistics for PVES Blocks*

Block	Description	M	SD	Min	Max	Skew	Kurt
1	Nude individuals without sexual activity	2.83	2.49	0	12	0.59	3.00
2	Non-aggressive sexual relations	4.55	2.18	0	12	0.58	4.83
3	Domination/submission without physical pain	2.80	2.93	0	16	1.19	4.57
4	Body fluid play (semen, saliva)	2.29	2.85	0	16	1.57	5.70
5	Sex with intoxicated/unconscious persons	0.82	2.15	0	16	3.47	16.13
6	Sexual relations with verbal threats/insults	0.74	1.96	0	15	3.65	18.36
7	Non-consensual sexual relations with force	0.89	2.08	0	16	3.23	15.17
8	Sexual relations with physical violence	1.40	2.31	0	16	2.45	10.57
9	Sexual relations with asphyxiation/strangulation	0.69	1.96	0	16	3.97	21.08
10	BDSM with implements (whips, wax, etc.)	1.13	2.27	0	16	2.82	12.51

Note: Blocks 1-2 have 3 items (range 0-12); Blocks 3-10 have 4 items (range 0-16). Kurtosis values represent raw kurtosis (normal distribution = 3.0). Sample: n = 2,181 participants with complete PVES data.

Table SM2b. *Descriptive Statistics for PVES Sub-Items*

Block	Sub-Item	N	M	SD	Skewness	Kurtosis
1	B6_1A	2181	1.23	1.44	0.80	2.20
1	B6_1B	2181	0.51	1.10	2.17	6.53
1	B6_1C	2181	1.09	1.25	0.91	2.73
2	B6_2A	2181	2.69	1.34	-0.74	2.35
2	B6_2B	2181	0.64	1.24	1.83	4.91
2	B6_2C	2181	1.23	1.22	0.75	2.59
3	B6_3A	2181	1.07	1.13	0.75	2.62
3	B6_3B	2181	0.40	0.88	2.38	8.10
3	B6_3C	2181	0.74	0.97	1.19	3.68
3	B6_3D	2181	0.59	0.91	1.53	4.75
4	B6_4A	2181	0.80	1.04	1.18	3.60
4	B6_4B	2181	0.37	0.87	2.50	8.58
4	B6_4C	2181	0.59	0.92	1.57	4.92
4	B6_4D	2181	0.52	0.90	1.86	6.08
5	B6_5A	2181	0.28	0.66	2.78	11.22
5	B6_5B	2181	0.15	0.56	4.49	24.22
5	B6_5C	2181	0.21	0.62	3.42	15.37
5	B6_5D	2181	0.19	0.59	3.69	17.49
6	B6_6A	2181	0.25	0.62	2.98	12.79
6	B6_6B	2181	0.13	0.50	4.41	24.14
6	B6_6C	2181	0.19	0.58	3.77	18.95
6	B6_6D	2181	0.17	0.55	4.06	21.52
7	B6_7A	2181	0.34	0.75	2.50	9.20
7	B6_7B	2181	0.14	0.54	4.36	23.42
7	B6_7C	2181	0.21	0.62	3.45	15.89
7	B6_7D	2181	0.19	0.58	3.53	16.47
8	B6_8A	2181	0.58	0.87	1.60	5.23
8	B6_8B	2181	0.20	0.62	3.66	17.29
8	B6_8C	2181	0.32	0.69	2.56	10.21
8	B6_8D	2181	0.30	0.68	2.75	11.43

9	B6_9A	2181	0.27	0.68	2.97	12.45
9	B6_9B	2181	0.11	0.48	5.26	33.53
9	B6_9C	2181	0.16	0.55	4.13	21.04
9	B6_9D	2181	0.16	0.56	4.46	25.11
10	B6_10A	2181	0.39	0.76	2.17	7.64
10	B6_10B	2181	0.19	0.61	3.87	18.97
10	B6_10C	2181	0.29	0.69	2.76	11.16
10	B6_10D	2181	0.26	0.65	2.97	12.52

Note: All sub-items scored on a 0–4 scale (0 = never, 4 = always). Sub-items grouped by block membership.

Sample: n = 2,181 participants with complete PVES data.

Table SM2c. *Spearman Correlations Among PVES Blocks*

	B1	B2	B3	B4	B5	B6	B7	B8	B9	B10
Block 1	—									
Block 2	.30	—								
Block 3	.25	.30	—							
Block 4	.35	.31	.53	—						
Block 5	.22	.14	.37	.38	—					
Block 6	.24	.16	.41	.40	.44	—				
Block 7	.17	.17	.43	.36	.53	.59	—			
Block 8	.19	.25	.46	.41	.35	.52	.47	—		
Block 9	.15	.16	.39	.35	.38	.50	.45	.56	—	
Block 10	.18	.23	.51	.40	.31	.46	.45	.53	.49	—

Note: Lower triangle shows correlations. All correlations significant at $p < .001$.

SM3. Empirical Justification for Item Parceling

This supplement provides the empirical analyses underpinning the parceling decision described in the main text. An exploratory factor analysis (EFA) of the 38 individual sub-items was conducted to verify whether the conceptual block structure of the PVES emerges as a dominant pattern in the data.

Method

The 38 sub-items (10 content blocks of 3–4 sub-items each) were submitted to EFA on the analytic sample of pornography consumers ($N = 2{,}181$). Maximum likelihood extraction was used with oblimin rotation, implemented through the *psych* package in *R*. The number of factors was determined by parallel analysis and the Kaiser criterion. Sampling adequacy was evaluated through the Kaiser–Meyer–Olkin (KMO) measure and Bartlett's test of sphericity. Items were considered to load substantially on a factor when $|\lambda| > .40$.

Results

Sampling adequacy and number of factors

Sampling adequacy supported the appropriateness of factor analysis: KMO = .94, with Bartlett's test of sphericity confirming significant intercorrelations, $\chi^2(703) = 67{,}704.5$, $p < .001$. The first five eigenvalues of the correlation matrix were 15.15, 4.13, 2.03, 1.76, and 1.46. Parallel analysis recommended the retention of 11 factors, while the Kaiser criterion (eigenvalue > 1) yielded 7. The 11-factor solution suggested by parallel analysis was retained for inspection.

Factor structure

Table SM3.1 displays items loading $|\lambda| > .40$ on each factor of the 11-factor EFA solution. Two coherent patterns emerged. First, seven factors aligned with single content blocks: ML3 with Block

5, ML1 with Block 6, ML5 with Block 7, ML10 with Block 8, ML6 with Block 9, ML8 with Block 10, and a joint factor ML4 covering Blocks 3 and 4. Second, two factors (ML2 and ML9) grouped sub-items "B" — those depicting same-sex male content — across Blocks 1–4 and Blocks 5–10, respectively.

Table SM3.1. *Items loading |λ| > .40 in the 11-factor EFA solution (maximum likelihood, oblimin rotation; factors with no items meeting threshold are not displayed)*

Factor	Items (ordered by loading)	Content domain
ML3	B6_5C (.87), B6_5A (.86), B6_5D (.79), B6_5B (.53)	Block 5
ML9	B6_6B (.68), B6_7B (.63), B6_9B (.62), B6_10B (.61), B6_8B (.60), B6_5B (.41)	Sub-items "B" — Blocks 5–10
ML4	B6_4C (.79), B6_4D (.74), B6_4A (.62), B6_3D (.45), B6_3C (.43)	Blocks 3–4
ML1	B6_6C (.78), B6_6A (.75), B6_6D (.72)	Block 6
ML2	B6_2B (.91), B6_1B (.83), B6_4B (.67), B6_3B (.55)	Sub-items "B" — Blocks 1–4
ML8	B6_10A (.74), B6_10C (.69), B6_10D (.67)	Block 10
ML5	B6_7A (.75), B6_7C (.70), B6_7D (.60)	Block 7
ML6	B6_9D (.69), B6_9C (.63), B6_9A (.60)	Block 9
ML10	B6_8C (.71), B6_8D (.61), B6_8A (.61)	Block 8

Note. Factor labels (ML1–ML11) are produced by the maximum-likelihood extraction routine and do not imply a substantive ordering. Standardized loadings are shown in parentheses.

SM4. Cross-Validation of the Optimized Bifactor Model

This supplement reports a 50/50 split-sample cross-validation of the optimized bifactor model presented in the main text. The objective is to evaluate the replicability of the model parameters and to test the equivalence of factor structure between two independent halves of the analytic sample.

Method

The analytic sample (n = 2,181) was randomly partitioned into two sub-samples of equal size, stratified by sex × age group: Sub-sample 1 (n = 1,090) and Sub-sample 2 (n = 1,091). Stratification yielded identical sex × age proportions across the two sub-samples (men 18–29: 358 vs 358; men 30–35: 338 vs 338; women 18–29: 210 vs 211; women 30–35: 184 vs 184).

The optimized bifactor model — with one general factor (G_violent) loading on all ten blocks, three specific factors loading on the corresponding content-level blocks (S_Level1: Blocks 1–4; S_Level2: Blocks 5–7; S_Level3: Blocks 8–10), strict orthogonality between G_violent and the specific factors and among the specific factors, and the two residual covariances retained from the specification search (full lavaan syntax in SM8) — was estimated separately on the total sample and on each sub-sample using maximum likelihood with robust standard errors and a scaled test statistic (MLR). The bifactor indices ECV, ωH, and PUC were computed for each sample. A multi-group analysis was then conducted across the two sub-samples to test configural, metric, and scalar invariance, applying the criteria of Chen (2007).

Results

Model fit

Table SM4.1 reports model fit indices for the optimized bifactor model in the total sample and in each sub-sample. Both sub-samples retained acceptable fit, with CFI > .97, RMSEA < .085, and SRMR < .03. Fit indices in Sub-sample 1 reproduced the values obtained in the total sample within narrow tolerance (ΔCFI = −.004; ΔRMSEA = +.005), while Sub-sample 2 exhibited marginally better fit (ΔCFI = +.008; ΔRMSEA = −.013).

Table SM4.1. *Model fit indices for the optimized bifactor model in the total sample and across cross-validation sub-samples*

Sample	*n*	χ^2 (scaled)	df	CFI	TLI	RMSEA [90% CI]	SRMR
Total	2,181	191.29	23	0.978	0.957	.076 [.066, .086]	0.025
Sub-sample 1	1,090	124.83	23	0.974	0.948	.081 [.068, .096]	0.029
Sub-sample 2	1,091	80.94	23	0.986	0.972	.063 [.048, .078]	0.021

Note. Estimator = MLR. Sub-samples obtained by random partitioning stratified by sex × age group.

Factor loadings

Tables SM4.2 and SM4.3 present the standardized factor loadings on G_violent and on the specific factors, respectively, in the total sample and in each sub-sample. Loadings on G_violent reproduced consistently across sub-samples, with a maximum absolute difference between sub-samples of .083 (at Block 2) and a mean absolute difference of .046. Loadings on the specific factors showed greater dispersion: the maximum absolute difference between sub-samples was .247 (at Block 8 on S_Level3), and three blocks (Block 5 on S_Level2, Block 7 on S_Level2, Block 8 on S_Level3) showed differences exceeding .15. This pattern is consistent with the variance-partitioning properties of bifactor models with a dominant general factor: when the general factor absorbs the bulk of the common variance (here, ECV = .81), the residual variance available to the specific factors is limited, and their loadings become more sensitive to sampling fluctuation.

Table SM4.2. *Standardized loadings on the general factor G_violent in the total sample and across cross-validation sub-samples.*

Block	λ Total	λ Sub-1	λ Sub-2
1	0.342	0.353	0.336
2	0.306	0.264	0.347
3	0.652	0.63	0.671
4	0.647	0.622	0.675
5	0.714	0.714	0.721
6	0.831	0.85	0.815
7	0.792	0.82	0.772
8	0.827	0.789	0.859
9	0.846	0.82	0.872
10	0.792	0.766	0.814

Table SM4.3. *Standardized loadings on the specific factors in the total sample and across cross-validation sub-samples*

Block	Specific factor	λ Total	λ Sub-1	λ Sub-2
1	S_Level1	0.295	0.246	0.351
2	S_Level1	0.340	0.304	0.380
3	S_Level1	0.395	0.438	0.352
4	S_Level1	0.517	0.529	0.504
5	S_Level2	0.310	0.184	0.403
6	S_Level2	0.312	0.335	0.309
7	S_Level2	0.355	0.258	0.415
8	S_Level3	0.309	0.411	0.164
9	S_Level3	0.185	0.211	0.171
10	S_Level3	0.212	0.280	0.149

Bifactor indices

Table SM4.4 reports ECV, ωH, and PUC across the three samples. The three indices were essentially identical across sub-samples (maximum absolute difference ≤ .014), supporting the stability of the variance-partitioning properties of the model.

Table SM4.4. *Bifactor indices in the total sample and across cross-validation sub-samples*

Index	Total	Sub-1	Sub-2
ECV	0.81	0.81	0.82
ωH	0.84	0.84	0.85
PUC	0.73	0.73	0.73

Note. ECV = explained common variance attributable to G_violent. ω_H = omega hierarchical for G violent. PUC = percentage of uncontaminated correlations.

Invariance between sub-samples

Multi-group analysis tested the equivalence of factor structure (configural model), factor loadings (metric model), and item intercepts (scalar model) between Sub-sample 1 and Sub-sample 2. As shown in Table SM4.5, both metric and scalar invariance were supported under the criteria of Chen (2007): from the configural to the metric model, ΔCFI = −.003 and ΔRMSEA = −.006; from the metric to the scalar model, ΔCFI = .000 and ΔRMSEA = −.003.

Table SM4.5. *Multi-group invariance analysis between cross-validation sub-samples*

Model	χ^2 (scaled)	df	CFI	TLI	RMSEA	SRMR	ΔCFI	ΔRMSEA
Configural	204.7	46	0.98	0.96	0.073	0.023	—	—
Metric	229.8	62	0.98	0.97	0.067	0.038	−.003	−.006
Scalar	242.6	68	0.98	0.97	0.064	0.038	.000	−.003

Note. Sub-sample 1 vs Sub-sample 2. Δ values reference the previous nested model. Criteria for invariance: ΔCFI ≤ .010 and ΔRMSEA ≤ .015 (Chen, 2007).

Conclusion

The optimized bifactor model replicates closely across two independent random halves of the analytic sample, stratified by sex × age. Loadings on G_violent reproduce within narrow sampling variation, the bifactor indices ECV, ωH and PUC remain virtually identical, and full scalar invariance between sub-samples is supported. Loadings on the specific factors show greater dispersion at three blocks, consistent with the residual nature of the specific factors in a bifactor model with a dominant general factor. Taken together, these results indicate that the structural properties of the model — and particularly the general factor used as the primary score — are robust to capitalization on chance.

SM5. Female-Victim and Male-Victim Sub-Score Analyses

This supplementary analysis disaggregates exposure to violent pornography by the gender configuration of the depicted victim, providing a sensitivity check on the predictive performance of the general factor G against alternative scoring schemes that retain victim-gender information.

Construction of Sub-Scores

Each item in Blocks 5, 6, 7, 8, 9, and 10 (the violent-content blocks) is structured as four sub-items reflecting actor–victim gender configurations: sub-item A (man toward woman), sub-item B (man toward man), sub-item C (woman toward man), and sub-item D (woman toward woman). Two sub-scores were constructed by summing across these blocks:

VAW (Violence Against Women): sum of the twelve sub-items with female victim (sub-items A and D): B6_5A, B6_5D, B6_6A, B6_6D, B6_7A, B6_7D, B6_8A, B6_8D, B6_9A, B6_9D, B6_10A, B6_10D.

VAM (Violence Against Men): sum of the twelve sub-items with male victim (sub-items B and C): B6_5B, B6_5C, B6_6B, B6_6C, B6_7B, B6_7C, B6_8B, B6_8C, B6_9B, B6_9C, B6_10B, B6_10C.

Sub-items were scored on a 0–4 scale (consistent with the recoded scale used throughout the manuscript and reported in SM2), yielding a theoretical range of [0, 48] for each sub-score (12 items × 4-point maximum). The analytic sample (n = 2,181) and predictor set (age, social desirability) match those reported in the manuscript.

Table SM5.1. *Descriptive Statistics*

Variable	*n*	*M*	*SD*	*Mdn*	IQR	Min	Max	Skew	Kurt
VAW	2,181	3.36	5.94	1.00	4.00	0.00	48.00	3.05	11.69
VAM	2,181	2.31	5.33	0.00	2.00	0.00	45.00	4.20	20.80
G factor	2,181	0.00	0.81	-0.28	0.51	-0.50	6.20	3.51	15.40

Table SM5.2. *Bivariate Correlations with 95% Bootstrap Confidence Intervals*

Pair	*r* (Pearson)	95% CI	*ρ* (Spearman)	95% CI
VAW × G	0.96	[.95, .96]	0.87	[.85, .88]
VAM × G	0.94	[.93, .95]	0.79	[.78, .81]
VAW × VAM	0.84	[.81, .87]	0.69	[.65, .71]

Note. Bias-corrected and accelerated bootstrap, 1,000 replications.

Predictive Performance for Sexual Violence Victimization (VS)

Table SM5.3. *Comparative Logistic Regression Models — Victimization*

Model	Predictor(s)	Nagelkerke R^2	Key OR [95% CI], *p*
Model G	G factor	0.102	2.00 [1.76, 2.28], $p < .001$
Model VAW	VAW	0.100	1.10 [1.08, 1.12], $p < .001$
Model VAM	VAM	0.094	1.11 [1.08, 1.13], $p < .001$
Model Both	VAW + VAM	0.105	VAW: 1.06 [1.03, 1.09], $p < .001$<br>VAM: 1.05 [1.01, 1.08], $p = .009$

Note. All models adjusted for age (A1) and social desirability (DSS).

Likelihood-ratio tests indicated that the combined model improved fit over both Model G ($\Delta\chi^2$ = 4.11, df = 1, $p = .043$) and Model VAW alone ($\Delta\chi^2$ = 7.04, df = 1, $p = .008$).

Table SM5.4. *Logistic Regression Coefficients — Combined Model (Victimization)*

Predictor	OR	95% CI	*p*
Intercept	1.03	[0.41, 2.54]	.956
A1 (age)	0.96	[0.93, 0.98]	.002
DSS	0.97	[0.93, 1.00]	.044
VAW	1.06	[1.03, 1.09]	< .001
VAM	1.05	[1.01, 1.08]	.009

Predictive Performance for Sexual Aggression (SA)

Table SM5.5. *Comparative Logistic Regression Models — Aggression*

Model	Predictor(s)	Nagelkerke R^2	Key OR [95% CI], *p*
Model G	G factor	0.205	2.68 [2.32, 3.13], $p < .001$
Model VAW	VAW	0.202	1.15 [1.12, 1.17], $p < .001$
Model VAM	VAM	0.199	1.15 [1.13, 1.18], $p < .001$
Model Both	VAW + VAM	0.214	VAW: 1.08 [1.04, 1.12], $p < .001$<br>VAM: 1.07 [1.03, 1.12], $p < .001$

Note. All models adjusted for age (A1) and social desirability (DSS).

Likelihood-ratio tests indicated that the combined model improved fit over both Model G ($\Delta\chi^2$ = 10.21, df = 1, $p = .001$) and Model VAW alone ($\Delta\chi^2$ = 12.72, df = 1, $p < .001$).

Table SM5.6. *Logistic Regression Coefficients — Combined Model (Aggression)*

Predictor	OR	95% CI	*p*
Intercept	0.09	[0.02, 0.39]	.001
A1 (age)	0.98	[0.94, 1.03]	.383
DSS	0.99	[0.93, 1.04]	.619
VAW	1.08	[1.04, 1.12]	< .001
VAM	1.07	[1.03, 1.12]	< .001

Summary

The two sub-scores capture extensively overlapping content: VAW and VAM correlate at $r = .84$, and both correlate near unity with the general factor ($r = .96$ and .94, respectively). Across both outcomes, the four scoring schemes yielded similar predictive performance (Nagelkerke r^2 ranging from .094 to .105 for victimization, and from .199 to .214 for aggression). The combined VAW + VAM model showed a small but statistically significant incremental gain over the general factor for victimization ($\Delta r^2 = .003$, $p = .043$) and for aggression ($\Delta r^2 = .009$, $p = .001$). Both sub-scores

contributed independent predictive variance in the combined models, but the magnitude of disaggregation effects was modest. These results provide convergent evidence that the general factor G captures the bulk of the predictive variance and remains the recommended primary score, while the disaggregated sub-scores can be reported when victim-gender configurations are theoretically central to a research question.

SM6. Distribution Analysis and Normality Assessment for PVES Block Scores

Table SM6. *Normality Tests for PVES Block Scores*

Block	Description	Shapiro W	*p*	K-S D	*p*	A-D A	Normal
1	Nude individuals without sexual activity	.903	<.001	.166	<.001	64.1	No
2	Non-aggressive sexual relations	.928	<.001	.169	<.001	54.0	No
3	Domination/submission without physical pain	.859	<.001	.170	<.001	85.5	No
4	Body fluid play (semen, saliva)	.794	<.001	.212	<.001	140.9	No
5	Sex with intoxicated/unconscious persons	.446	<.001	.429	<.001	492.4	No
6	Sexual relations with verbal threats/insults	.440	<.001	.442	<.001	495.1	No
7	Non-consensual sexual relations with force	.496	<.001	.417	<.001	440.2	No
8	Sexual relations with physical violence	.662	<.001	.294	<.001	248.1	No
9	Sexual relations with asphyxiation/strangulation	.411	<.001	.444	<.001	516.2	No
10	BDSM with implements (whips, wax, etc.)	.569	<.001	.375	<.001	359.7	No

Note: Shapiro W = Shapiro-Wilk test statistic; K-S D = Kolmogorov-Smirnov test statistic; A-D A = Anderson-Darling test statistic. All tests evaluate departure from normal distribution, with significant p-values ($p < .05$) indicating non-normality. Normal = classification based on combined evidence from all three tests (No = significant departure from normality).

Summary by Content Level

Content Type	Distribution Pattern	Normality Result
Level 1 baseline (Blocks 1-2)	Relatively symmetric with moderate skewness	All tests reject ($p < .001$)
Level 1 transitional (Blocks 3-4)	Moderate positive skew	All tests reject ($p < .001$)
Level 2 (Blocks 5-7)	Positive skew with floor effects	All tests reject ($p < .001$)
Level 3 (Blocks 8-10)	Severe positive skew with extreme floor effects	All tests reject ($p < .001$)

Figure SM6.1. Histograms of PVES Block Score Distributions

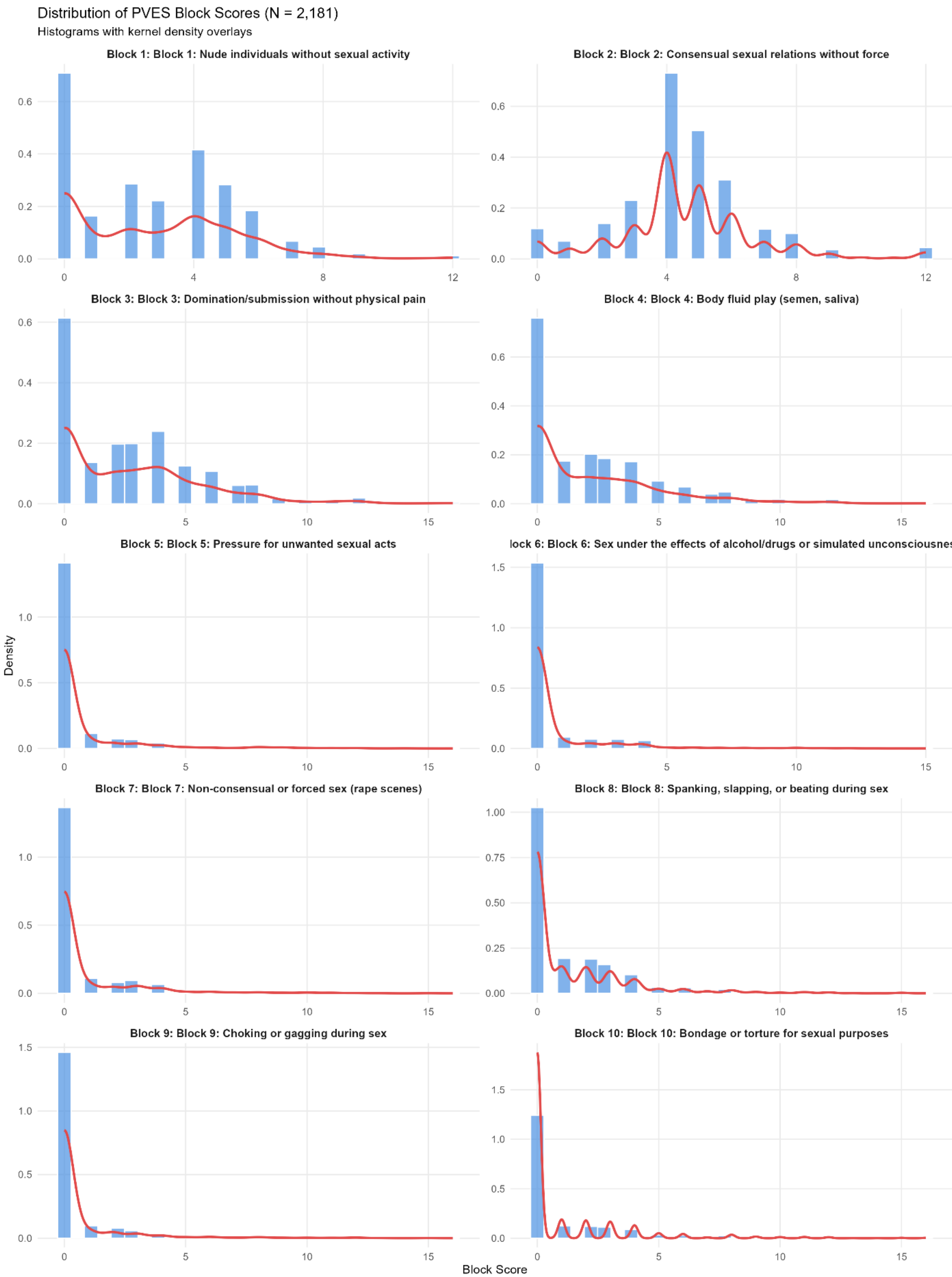

Figure SM6.2. Q-Q Plots for PVES Block Scores

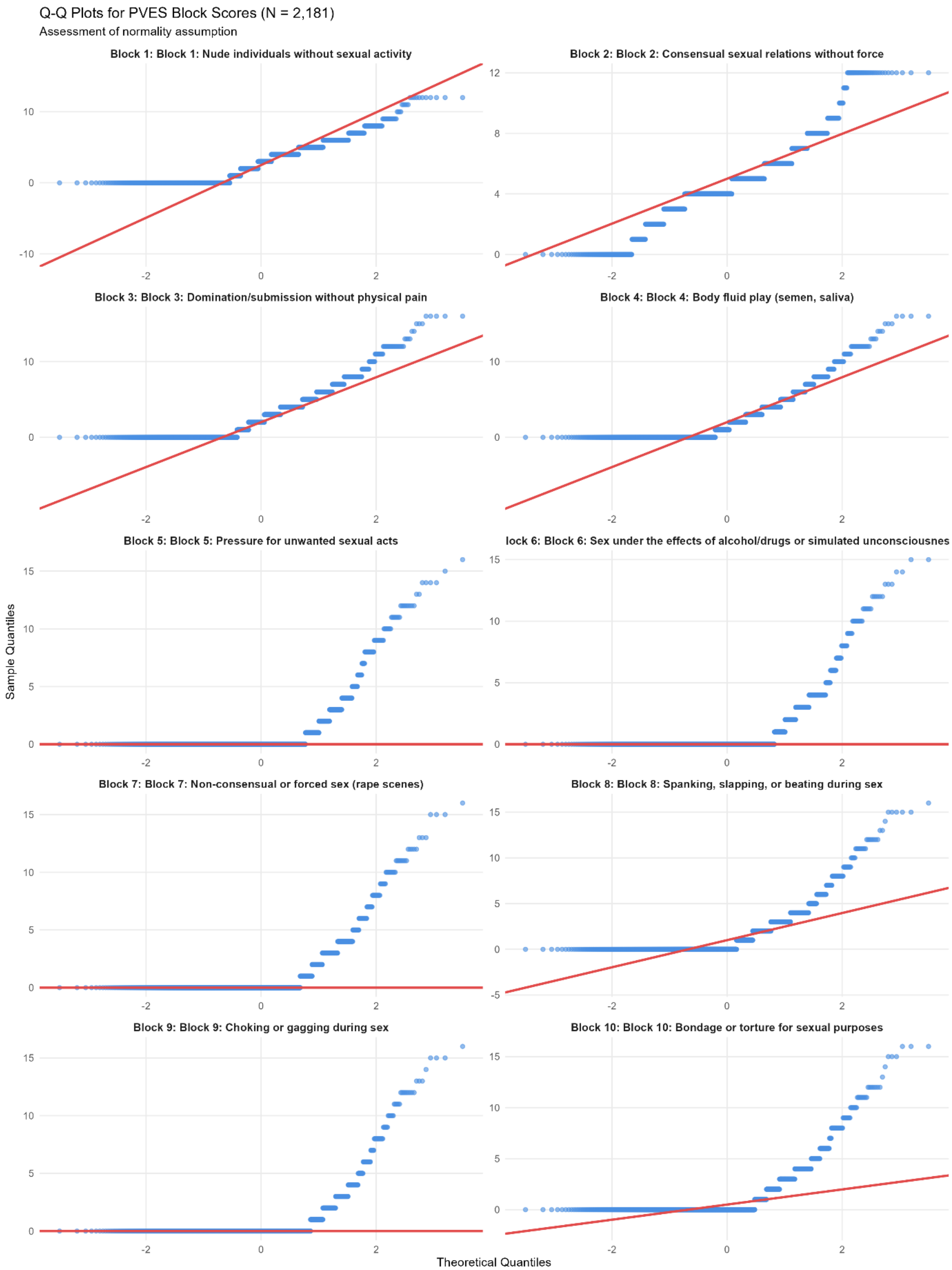

SM7. Factor G Specificity Analysis: Detailed Validation Evidence

This supplementary analysis provides granular evidence that Factor G from the optimized bifactor model specifically measures exposure to violent pornographic content rather than general consumption variability. The analysis examines correlations between Factor G and individual content blocks, as well as factor-based content aggregations.

Table SM7.1. *Individual Block Correlations with Factor G*

Block	Factor	Description	Level	*r*
9	F3	Relations with asphyxiation/strangulation	Level 3	.892
6	F2	Relations with threats/verbal insults	Level 2	.875
8	F3	Relations with physical aggression	Level 3	.871
10	F3	BDSM with implements	Level 3	.835
7	F2	Non-consensual relations with force	Level 2	.835
5	F2	Relations with intoxicated/unconscious persons	Level 2	.752
3	F1	Domination/submission without physical pain	Level 1 (transitional)	.687
4	F1	Games with body fluids	Level 1 (transitional)	.679
1	F1	Nude persons without sexual activity	Level 1 (baseline)	.360
2	F1	Consensual sexual relations	Level 1 (baseline)	.323

Note. n = 2,181. Pearson correlations; all significant at $p < .001$.

The correlation pattern demonstrates a clear gradient from Level 1 baseline content (r = .323–.360) through Level 1 transitional content (r = .679–.687) and Level 2 content (r = .752–.875) to Level 3 content (r = .835–.892).

Table SM7.2. *Factor G Correlations with Content Aggregations*

Content Aggregation	*r* with Factor G	Interpretation
F3 (Level 3)	.955	Highest correlation
F2 (Level 2)	.911	Highest correlation
Level 1 transitional (Blocks 3-4)	.756	Moderate-high correlation
F1 (Level 1)	.709	Moderate-high correlation
Level 1 baseline (Blocks 1-2)	.418	Lowest correlation

Note. Pearson correlations; n = 2,181.

Specificity Evidence

a) Level 3 vs. Level 1 baseline difference: Δr = .537
b) Level 2 vs. Level 1 baseline difference: Δr = .493
c) Level 1 transitional vs. Level 1 baseline difference: Δr = .337

All differences exceed the .30 threshold for meaningful specificity, with Level 2 and Level 3 content showing substantially larger effect sizes.

Table SM7.3. *Consumption Progression Typology and Factor G Distribution*

Typology	n	%	M(G)	SD(G)	% G < 0
Low general consumption	1145	52.5	-0.399	0.100	100.0
Level 1 only	170	7.8	-0.231	0.123	97.1
Up to Level 2	283	13.0	-0.026	0.284	54.4
Up to Level 3	583	26.7	0.864	1.139	18.9

Note: High consumption defined as ≥75th percentile for each factor.

The progression shows systematic increases in Factor G scores, with individuals consuming only Level 1 content maintaining negative Factor G scores in 97.1% of cases.

Table SM7.4. *Consumption Typology Distribution by Factor G Tertiles*

Typology	Low Tertile	Medium Tertile	High Tertile
Low general consumption	61.5%	37.9%	0.6%
Level 1 only	8.2%	79.4%	12.4%
Up to Level 2	3.2%	44.5%	52.3%
Up to Level 3	0.0%	5.5%	94.5%

The distribution demonstrates perfect progression patterns within each consumption typology: individuals with low general consumption are predominantly found in low tertiles (61.5% low, only 0.6% high), while those consuming up to Level 3 are overwhelmingly concentrated in the high tertile (94.5%). Level 1 only consumers cluster primarily in the medium tertile (79.4%), and those consuming up to Level 2 show an ascending distribution across tertiles (3.2% → 44.5% → 52.3%). This ordinal progression within each typology provides strong evidence that Factor G specifically measures violent content exposure rather than general consumption variability.

Methodological Validation Summary

Criterion	Threshold	Obtained	Result
Level 3 vs. Level 1 baseline difference	> 0.30	0.537	✓ Excellent
Level 2 vs. Level 1 baseline difference	> 0.30	0.493	✓ Excellent
Level 1 only group Factor G < 0	< 0	-0.231	✓ Excellent
Coherent gradient across content levels	Progressive	Yes	✓ Perfect

Tertile ordering by typology	Ordinal	Yes	✓ Perfect

Table SM7.5. *Correlation Between Factor G and Self-Reported Frequency of Online Pornography Viewing*

Coefficient	*n*	Estimate	BCa 95% CI
Spearman ρ	2,153	.342	[.303, .381]
Pearson r	2,153	.226	[.186, .264]

Note. Criterion: self-reported frequency of viewing online pornography in the past 12 months (8-point item); n = participants with a valid response. BCa = bias-corrected and accelerated bootstrap confidence interval (2,000 replications).

Self-reported frequency of viewing online pornography in the past 12 months (8-point item) was available for 2,153 participants. The general factor correlated moderately with this indicator: Spearman ρ = .34, BCa 95% CI [.30, .38]; Pearson r = .23, BCa 95% CI [.19, .26] (2,000 bootstrap replications). Because the PVES item stem is conditional on the viewing occasion, this moderate association is consistent with the general factor indexing the content viewed rather than the amount of viewing.

Conclusion

All five stringent criteria for specificity are met with large effect sizes, providing compelling evidence that Factor G measures exposure to violent pornographic content specifically, rather than general consumption variability. The granular analysis provides converging evidence from multiple analytical approaches: block-level correlations demonstrate a systematic gradient from Level 1 baseline (.323–.360) to Level 3 content (.835–.892); factor-based aggregations show large differences between Level 2-3 and Level 1 content ($\Delta r > .490$); consumption typology reveals that

individuals with high Level 1-only consumption maintain negative Factor G scores; and tertile distributions demonstrate perfect ordinality in the progression toward Level 2 and Level 3 content. These findings establish that Factor G represents a specific measure of violent pornography exposure, supporting its theoretical interpretation and practical application in research on pornography and sexual violence.

SM8. R lavaan Syntax for the Optimized Bifactor Model

This section provides the lavaan model syntax used to estimate the optimized bifactor specification reported in Results (Confirmatory Factor Analysis). The syntax is presented without estimated parameters to facilitate technical reproduction.

Block scores (`B6_bloc01` to `B6_bloc10`) were computed as the sum of the sub-items belonging to each block (see Table SM2b for the sub-item-to-block mapping).

Model syntax

```r

bifactor_model <- '
  # General factor
  G_violent =~ B6_bloc01 + B6_bloc02 + B6_bloc03 + B6_bloc04 +
               B6_bloc05 + B6_bloc06 + B6_bloc07 +
               B6_bloc08 + B6_bloc09 + B6_bloc10

  # Specific factors by content level
  S_Level1 =~ B6_bloc01 + B6_bloc02 + B6_bloc03 + B6_bloc04
  S_Level2 =~ B6_bloc05 + B6_bloc06 + B6_bloc07
  S_Level3 =~ B6_bloc08 + B6_bloc09 + B6_bloc10

  # Orthogonality constraints (strict bifactor)
  G_violent ~~ 0*S_Level1 + 0*S_Level2 + 0*S_Level3
  S_Level1  ~~ 0*S_Level2 + 0*S_Level3
  S_Level2  ~~ 0*S_Level3

  # Residual covariances retained after specification search
```

```
  B6_bloc01 ~~ B6_bloc02

  B6_bloc04 ~~ B6_bloc05
'
```

Estimation call

```r

fit_bifactor <- cfa(bifactor_model, data = porndf, estimator = "MLR")

```

Notes

Estimation employed maximum likelihood with robust standard errors and a scaled test statistic (MLR), consistent with the analyses reported in the manuscript. Strict orthogonality between the general factor and the specific factors, and among the specific factors themselves, was imposed to facilitate variance partitioning through ECV, ωH, and PUC indices. The two residual covariances were retained from the specification search reported in Results based on substantive content overlap between adjacent blocks at the same content level (Blocks 1–2 within Level 1 baseline; Blocks 4–5 at the Level 1-transitional/Level 2 boundary).

SM9. Sensitivity of Criterion Validity to Response Quality

A small number of respondents showed response patterns suggestive of inattentive or indiscriminate responding on the sexual violence items, identified a priori by three criteria applied to the full survey sample ($N = 3{,}607$): (1) reporting no lifetime sexual contact (or preferring not to answer) while endorsing any perpetration item; (2) extreme straight-lining across all 80 SAV-S items (all responses in the maximum category); (3) total completion time below one third of the median. In the analytic sample ($n = 2{,}181$), 15 participants met at least one criterion (14 by criterion 1, 1 by criterion 2, none by criterion 3). Table SM9.1 compares the convergent correlations (Table 4) and the Model 2 incremental validity estimates (Table 5) with and without these participants. All estimates were essentially unchanged.

Table SM9.1. *Convergent correlations and Model 2 incremental validity estimates with and without respondents flagged for response quality*

A. Convergent correlations (Spearman) with 95% confidence intervals

	Full sample (n = 2,181)		Excluding flagged (n = 2,166)	
Variable	r	95% CI	r	95% CI
Sexual victimization: partner	.23	[.19, .27]	.22	[.18, .26]
Sexual victimization: friend or acquaintance	.25	[.21, .29]	.24	[.19, .28]
Sexual victimization: stranger	.22	[.18, .26]	.21	[.17, .25]
Sexual perpetration: partner	.26	[.22, .30]	.25	[.21, .29]
Sexual perpetration: friend or acquaintance	.26	[.22, .30]	.25	[.21, .29]
Sexual perpetration: stranger	.24	[.20, .28]	.23	[.18, .27]
Sextortion threats: show	.21	[.17, .25]	.20	[.15, .24]
Sextortion threats: publish	.22	[.18, .26]	.21	[.16, .25]

B. Incremental validity: Model 2 estimates

Outcome	Full sample (n = 2,181)	Excluding flagged (n = 2,166)

	Nagelkerke R^2 (Model 1 → Model 2)	ΔR^2	*OR* [95% CI]	Nagelkerke R^2 (Model 1 → Model 2)	ΔR^2	*OR* [95% CI]
Sexual victimization	.014 → .102	.088	2.00 [1.76, 2.28]	.012 → .094	.081	1.96 [1.72, 2.24]
Sexual perpetration	.004 → .205	.200	2.68 [2.32, 3.13]	.002 → .199	.197	2.69 [2.32, 3.15]

Note. Flagged respondents (*n* = 15) met at least one a priori criterion: no lifetime sexual contact (or no answer) combined with endorsed perpetration (*n* = 14); extreme straight-lining across all 80 SAV-S items (*n* = 1); completion time below one third of the median (*n* = 0). Correlations are Spearman coefficients with Fisher 95% confidence intervals. Model 1: age + social desirability; Model 2: + PVES general factor. Odds ratios with profile-likelihood 95% confidence intervals.
